# The Impact of T-cell Exhaustion Dynamics on Tumour–Immune Interactions and Tumour Growth

**DOI:** 10.1007/s11538-025-01433-1

**Published:** 2025-04-02

**Authors:** Nicholas Lai, Alexis Farman, Helen M. Byrne

**Affiliations:** 1 Wolfson Centre for Mathematical Biology, Mathematical Institute, University of Oxford, Oxford, OX2 6GG UK; 2 Department of Mathematics, University College London, London, WC1E 6BT UK; 3 Ludwig Institute for Cancer Research, University of Oxford, Oxford, OX3 7DQ UK

**Keywords:** Cancer, Immunology, T-cells, Exhaustion, Structured mathematical model

## Abstract

Tumours evade immune surveillance through a number of different immunosuppressive mechanisms. One such mechanism causes cytotoxic T-cells, a major driving force of the immune system, to differentiate to a state of ‘exhaustion’, rendering them less effective at killing tumour cells. We present a structured mathematical model that focuses on T-cell exhaustion and its effect on tumour growth. We compartmentalise cytotoxic T-cells into discrete subgroups based on their exhaustion level, which affects their ability to kill tumour cells. We show that the model reduces to a simpler system of ordinary differential equations (ODEs) that describes the time evolution of the total number of T-cells, their mean exhaustion level and the total number of tumour cells. Numerical simulations of the model equations reveal how the exhaustion distribution of T-cells changes over time and how it influences the tumour’s growth dynamics. Complementary bifurcation analysis shows how altering key parameters significantly reduces the tumour burden, highlighting exhaustion as a promising target for immunotherapy. Finally, we derive a continuum approximation of the discrete ODE model, which admits analytical solutions that provide complementary insight into T-cell exhaustion dynamics and their effect on tumour growth.

## Introduction

Cancer is a complex and multi-faceted disease. Our understanding of the mechanisms that drive its progression is constantly improving. For example, recent developments are unravelling the multitude of factors at play within the tumour micro-environment and, in particular, the importance of tumour-immune interactions in shaping cancer evolution (Junttila and De Sauvage 2013).

The tumour-microenvironment (TME) refers to the complex network of cells and stroma that surround a tumour (Hanna et al. 2009). These include endothelial cells, pericytes, fibroblasts, immune cells and extracellular matrix. The TME evolves continuously during tumour growth, with cross-talk between the different cell types regulating tumour progression (Elmusrati et al. 2021). Within the TME, the immune landscape—comprising T-cells, B-cells, macrophages, neutrophils, and others—has a huge impact on whether a tumour regresses or progresses. The primary role of the immune system is to defend the body against infection and protect a host’s cells. Cancer, however, often reprogrammes the immune system so that, rather than destroying cancer cells, it promotes tumour progression. Understanding and quantifying the mechanisms by which cancer evades immune surveillance is vital for further improvement in treatment.

In practice, the immune system facilitates both tumour suppression and progression, and the balance between these opposing effects determines whether or not a tumour grows. To capture the qualitative behaviours that can occur, Schreiber et al. proposed ‘the three E’s of cancer immunoediting’ - elimination, equilibrium and escape (Dunn et al. 2004). ‘Elimination’ occurs when the immune system successfully kills all of the tumour cells; ‘equilibrium’ occurs when the suppressive effects of the immune system balance the tumour’s growth, maintaining it at a fixed size; ‘escape’ occurs when the suppressive effects of the immune system are unable to control the tumour’s growth. Immunotherapy aims to bolster the immune response and mitigate the immunosuppressive effects of cancer in order to achieve ‘tumour elimination’.

Over the last 40 years, increased understanding of tumour-immune interactions has stimulated the development of a range of immunotherapeutic treatments for cancer (Farkona et al. 2016). These include checkpoint inhibitors which block proteins that suppress immune-cell activity, and CAR T-cell therapy where a patient’s T-cells are genetically altered to more effectively locate and kill cancer cells (Tabana et al. 2021; Sterner and Sterner 2021). In clinical trials (Egen et al. 2020; Kang et al. 2016), immunotherapies have been administered alone and in combination with other treatments, such as radiotherapy and chemotherapy, yielding marked improvements in patient survival rates and quality of life compared with the current standard of care (Esfahani et al. 2020; Hall et al. 2013; Smyth et al. 2016). However, immunotherapy has limitations. It is difficult to predict the efficacy of immunotherapeutic treatments, due to large variations in patient responses. Additionally, treatment can sometimes induce an inflammatory response against the host’s healthy tissue rather than the tumour (Taefehshokr et al. 2022; Esfahani et al. 2020). In order to improve the efficacy of immunotherapy we require a better understanding of the mechanisms by which tumours evade and/or reprogram the immune system.

As mentioned above, T-cells play a major role in the immune response against cancer. There are many subtypes, including helper T-cells and regulatory T-cells. Of particular importance here are cytotoxic T-cells which locate and kill cancer cells. Within the TME, chronic stimulation by tumour antigens causes cytotoxic T-cells to differentiate to a hyporesponsive state of ‘exhaustion’. T-cell exhaustion represents a dysfunctional state in which cytotoxic T-cells express increased levels of inhibitory receptors, such as PD-1 and CTLA-4, which reduce their ability to recognise and kill tumour cells (Jiang et al. 2015; Blank et al. 2019). These behavioural changes reduce the immune system’s ability to suppress the tumour and are correlated with poor patient-outcome in multiple cancer types (e.g., head and neck cancer, renal cell carcinoma, breast cancer, etc.) (Chow et al. 2022; Kansy et al. 2017; Thompson et al. 2007; Sun et al. 2014; Muenst et al. 2013). A number of exhaustion states have been identified, ranging from precursor-exhausted T-cells with stem-like properties to terminally differentiated T-cells that exhibit a complete loss of effector function (Dolina et al. 2021). However, it is now understood that T-cell exhaustion is a continuous process that spans a spectrum of exhaustion states, along which T-cells differentiate while progressively losing effector function (Chow et al. 2022). If immunotherapy is to realise its full potential, we must first better understand how T-cell exhaustion impacts immune evasion and explore treatments that mitigate its adverse effects (Chow et al. 2022). In this study, we develop a mathematical model to investigate how exhaustion inhibits the ability of cytotoxic T-cells to kill tumour cells and the extent to which T-cell exhaustion may contribute to the 3 E’s of immunoediting.

Mathematical modelling represents a valuable tool to help oncologists and clinicians solve the complex puzzle that is cancer. When combined with experiments, mathematical models can be used to generate and test hypotheses, and achieve a level of understanding that surpasses what can be obtained using each approach in isolation (Byrne et al. 2006). A variety of mathematical approaches have been used to model the growth and response to treatment of solid tumours: these include ordinary differential equations (ODEs), partial differential equations (PDEs), stochastic models, agent-based models (ABMs), and hybrid models (further details can be found in the following review articles (Eftimie et al. 2011; Enderling and AJ Chaplain 2014; Bull and Byrne 2022; Rejniak and Anderson 2011; Yin et al. 2019)). Many mathematical models of tumour-immune interactions are formulated as time-dependent ODEs. Of these, Kuznetsov et al.’s seminal model serves as a benchmark.  Kuznetsov et al. (1994) use a predator–prey approach, treating the immune system as a single, generic species and comparing their model with experimental data. Many authors have extended Kuznetsov’s model to investigate the effect of time-delays in immune cell recruitment (Khajanchi and Banerjee 2014), the impact of chemotherapy (Kiran and Lakshminarayanan 2013), and the effects of both the innate and adaptive immune responses and associated cytokines (Pillis and Radunskaya 2003; Robertson-Tessi et al. 2012). Detailed bifurcation and asymptotic analyses have also been performed and have demonstrated that Kuznetsov’s original model exhibits excitable dynamics  (Dritschel et al. 2018; Osojnik et al. 2020).

Additional complexity arises due to phenotypic heterogeneity within a particular cell type. For example, macrophages exhibit phenotypic heterogeneity as they transition between pro-inflammatory, phagocytic, M1-like macrophages and anti-inflammatory M2-like macrophages (Najafi et al. 2019) and cytotoxic T-cells exhibit heterogeneity in their exhaustion status (Jiang et al. 2015). Several authors have developed structured mathematical models to investigate the effect that heterogeneity has on tumour growth. For example, cancer cells have been structured by age (Bekkal Brikci et al. 2008), expression of drug-resistance genes (Lorz et al. 2013; Stace et al. 2020), cell-cycle position and/or cell-cycle protein content (Hodgkinson et al. 2023; Basse et al. 2004), and stemness (Celora et al. 2021). While most structured models focus on a single structure variable, several authors have proposed dual-structured models, to account for spatial and phenotypic heterogeneity in cancer cells (Celora et al. 2023; Villa et al. 2021; Hodgkinson et al. 2019; Lorz et al. 2015). By contrast, Bartha and Eftimie (2022) propose a dual-structured model of macrophage-tumour interactions which accounts for spatial heterogeneity and where macrophages are structured by their phenotype. Mathematical models that account for different types of heterogeneity provide a nuanced understanding of tumour-immune interactions and may reveal complex emergent behaviours not captured by simpler homogenous models.

T-cell exhaustion has been incorporated into several mathematical models. For example, Kareva and Gevertz (2024) explicitly model populations of ‘reversibly exhausted’ and ‘terminally exhausted’ T-cells using a system of ODEs, where immune checkpoint blockade only affects the former, and explore how the model behaves for different treatment protocols. Sahoo et al. (2020) formulate an ODE model that describes how CAR T-cells and tumour-cells interact, and use it to explore the potential of T-cell exhaustion to inhibit CAR T-cell therapy. In practice, most existing models of T-cell exhaustion focus on a small number of discrete exhaustion states. However, T-cell exhaustion is now understood to be a dynamic process, spanning a spectrum of exhaustion states, with T-cells progressively losing effector function as they become more exhausted (Jiang et al. 2021; Chow et al. 2022). We aim to study this process and investigate its effect on tumour growth in a mathematical model where T-cells are structured by their exhaustion status.

In this paper, we present three, related mathematical models of T-cell exhaustion in cancer: (i) a discrete ODE model, in which the T-cells are structured by their exhaustion level; (ii) a reduced ODE model describing the time evolution of the total number of T-cells, their mean exhaustion level, and the total number of tumour cells, obtained by taking moments of the discrete model; (iii) a PDE model obtained by treating T-cell exhaustion as a continuous variable and taking a continuum approximation of the discrete ODE model. We use these models to investigate how the exhaustion distribution of the T-cells changes over time due to interactions with the tumour cells and how these changes, in turn, affect the tumour’s growth dynamics. We identify distinct T-cell distributions with the tumour’s long-term behaviour. In particular, tumour elimination is associated with a T-cell distribution which is skewed towards highly active T-cells, tumour escape is associated with a distribution that is skewed towards exhausted T-cells, and tumour equilibrium is associated with a T-cell population that is more uniformly distributed across the exhaustion spectrum. The discrete ODE model and the PDE model are novel in that they are the first structured models to investigate the impact of T-cell exhaustion on tumour growth. While ODE models investigating tumour-immune interactions have been proposed, the novelty of the reduced ODE model is that it is derived from a structured model and, as such, characterises the T-cells in terms of their number and mean exhaustion level based on a fundamental description of the biology. Additionally, comparison of the discrete ODE model with the reduced ODE model shows how parameters at a discrete scale coarse grain to macro and population scale.

The remainder of this paper is organised as follows. In Sect. 2, we propose a spatially-averaged, time-dependent mathematical model of tumour-immune interactions, in which T-cells are structured by their exhaustion level. We show how the model can be reduced to a simpler system of ODEs describing the time evolution of the total number of T-cells, their mean exhaustion level, and the total number of tumour cells. We then non-dimensionalise the model equations and present parameter values retrieved from the literature. In Sect. 3, we present numerical simulations showing the coevolution of T-cells and tumour cells for different parameter values, recapitulating the 3 E’s of immunoediting and illustrating the T-cell exhaustion distribution in each case. We then solve the steady states of the system and characterise their linear stability. We conduct bifurcation analysis and identify distinct regions of parameter space in which we expect to see these different qualitative behaviours and where the system exhibits bistability. We also identify parameter values for which the system exhibits tristability and all 3 E’s of immunoediting are simultaneously stable. Finally, we present numerical simulations demonstrating the impact of immunotherapy on model dynamics. In Sect. 4, we propose a PDE model of T-cell exhaustion dynamics, derived from the discrete ODE model by treating a T-cell’s exhaustion level as a continuous variable. The PDE model admits analytical solutions, which we compare with solutions to the discrete model.

## Model Development

In this section, we introduce a mathematical model that describes how T-cell exhaustion and interactions with tumour cells impact the growth dynamics of the tumour cells. For simplicity, spatial effects are neglected. We compartmentalise the cytotoxic T-cell population into distinct classes, based on their exhaustion status. Their time-evolution is governed by a system of time-dependent ordinary differential equations (ODEs) which are coupled to an ODE describing the time-evolution of the number of tumour cells. After introducing the governing equations, we show how the model can be reduced to a closed system of ODEs describing the time evolution of the total number of T-cells, their mean exhaustion level, and the total number of tumour cells.

**Fig. 1:**
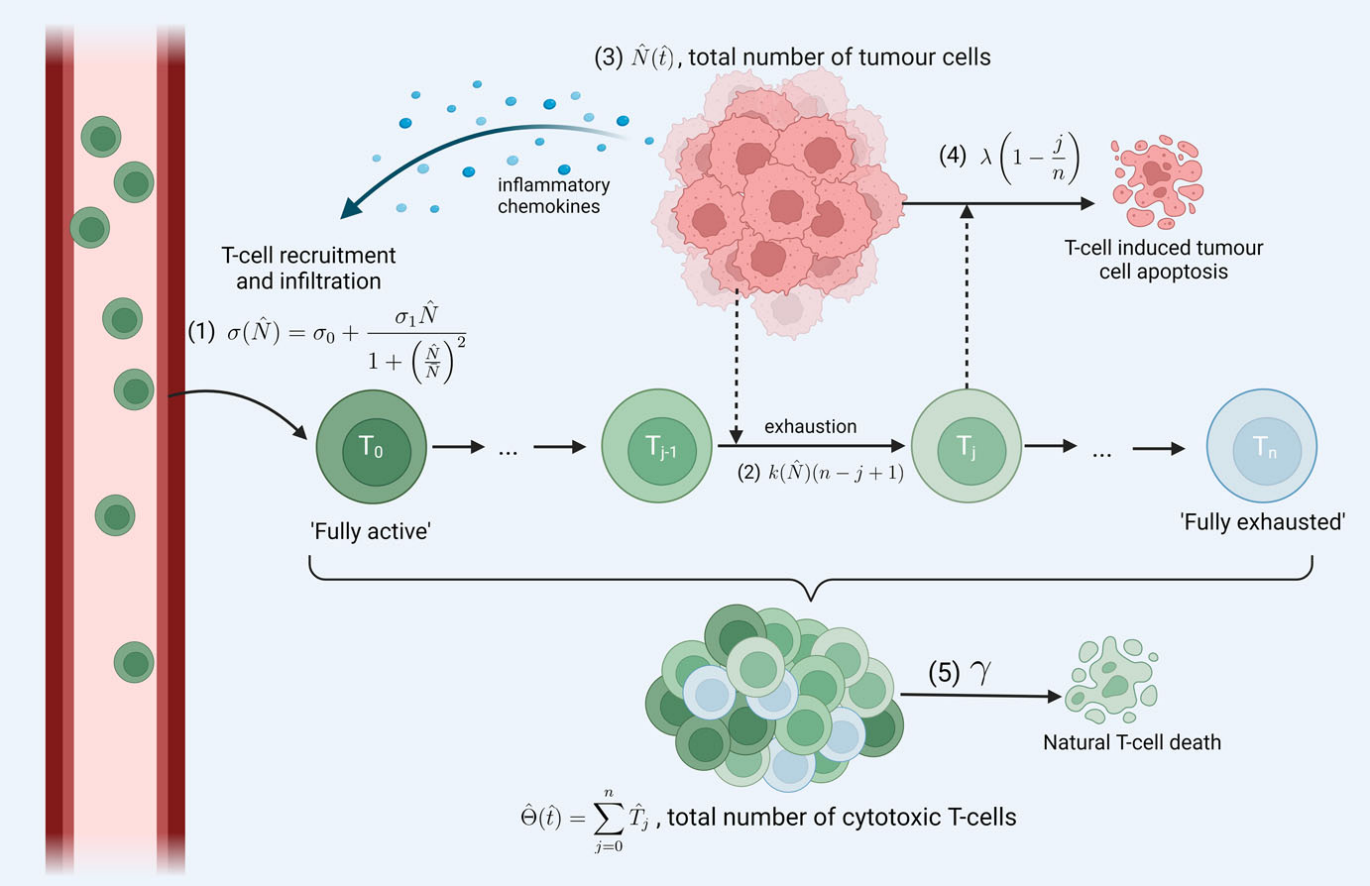
Model schematic. Diagram of the model setup, consisting of a tumour cell population (*N*(*t*)), and a T-cell population compartmentalised into $n+1$ subgroups ($T_j(t), j=0,1, \ldots , n$) according to their exhaustion level, *j*. The system dynamics are dominated by the following processes: (1) influx of T-cells into the tumour microenvironment in response to chemokines released by the tumour cells; (2) progressive T-cell exhaustion due to interactions with the tumour cells; (3) logistic growth of the tumour cells; (4) T-cell induced death of tumour cells at a rate which depends on the T-cell’s exhaustion level; (5) natural death of T-cells. Created with https://www.biorender.com/ (Color figure online)

### Model Derivation

We consider populations of cytotoxic T-cells and tumour cells, and their interactions within the tumour microenvironment (see Fig. 1). While existing models of T-cell exhaustion incorporate a small number of exhaustion states, T-cell exhaustion is now understood to span a spectrum of exhaustion states, along which T-cells differentiate while progressively lose effector function (Jiang et al. 2021; Chow et al. 2022; Dolina et al. 2021). Therefore, we propose a structured model of exhaustion in which we decompose the T-cell population into $n+1$ compartments according to their exhaustion level *j*, where $j \in \{ 0,1,\ldots ,n \}$. We denote by $\hat{T}_j(\hat{t})$ the number of cytotoxic T-cells at time $\hat{t}$ with exhaustion level *j*, where $\hat{T}_{j=0}(\hat{t})$ and $\hat{T}_{j=n}(\hat{t})$ denote the numbers of fully active and fully exhausted cytotoxic T-cells, respectively. While different exhaustion states may have diverse characteristics, for simplicity, we assume a T-cell’s exhaustion level only influences its ability to kill tumour cells and its exhaustion rate. We denote by $\hat{N}(\hat{t})$ the number of tumour cells at time, $\hat{t}$. We model their co-evolution with the following equations:123We will refer to equations (1)–(3) as the **discrete ODE model**. In equation (1), we assume that when T-cells are recruited to the TME they are fully active (i.e., $j=0$). We assume further that two terms contribute to the rate of T-cell influx: a constant, or basal, supply rate $\sigma _0$, and an additional term due to tumour-derived cytokines which, for simplicity, we assume depends biphasically on $\hat{N}$. Thus, we assume that when the tumour is small, the rate of T-cell infiltration increases approximately linearly with $\hat{N}$, with constant of proportionality $\sigma _1$, due to increased rates of production of pro-inflammatory chemokines and cytokines by the tumour and surrounding stroma. As the tumour increases in size, it inhibits immune cell infiltration into the TME (Joyce and Fearon 2015). We account for this effect via the factor of $\left( 1 + \left( \frac{\hat{N}}{\bar{N}} \right) ^2 \right) ^{-1}$ which ensures that the tumour-stimulated influx decreases to zero as $\hat{N} \rightarrow \infty$. The parameter $\bar{N}$ denotes the number of tumour cells at which the T-cell influx is maximised. We note that similar functional forms have been used by other authors (Kuznetsov et al. 1994; Dritschel et al. 2018).

We model T-cell exhaustion via the reaction:

$$ \hat{T}_{j} \xrightarrow {\hat{k}(\hat{N})n \left( 1 - \frac{j}{n} \right) } \hat{T}_{j+1}, \qquad \qquad j = 0,1,...,n-1. $$

Here, we assume that the exhaustion rate depends on the number of tumour cells, $\hat{N}$, via the increasing function $\hat{k}(\hat{N})$, since exhaustion is primarily due to overstimulation of T-cells due to chronically high tumour-antigen load (Blank et al. 2019). For simplicity, we assume that this function is linear, $\hat{k}(\hat{N}) = k_{0} + k_{1}\hat{N}$, where $k_0$ is the basal exhaustion rate and $k_{1}\hat{N}$ is the increase in this rate due to the presence of tumour cells. We further assume that the rate at which T-cells become exhausted is a linearly decreasing function of their exhaustion level, *j*, since exhaustion upregulates inhibitory receptors and reduces effector function which, in turn, reduces the rate of further stimulation by tumour antigens (Saka et al. 2020). We scale the exhaustion rate by a factor of *n* to ensure that the average time to transition from ‘fully active’ to ‘fully exhausted’ is independent of the number of compartments used to structure the T-cell population. We also assume that all T-cells undergo natural cell death at a constant rate, $\gamma$.

We suppose that the evolution of the tumour cell population is dominated by cell proliferation and T-cell induced cell death. We assume that, in the absence of T-cells, tumour cells undergo logistic growth, with growth rate *r* and carrying capacity *K*. We note that as T-cells become more exhausted, they express an increased number of inhibitory receptors and exhibit impaired cytotoxicity (Jiang et al. 2015). Therefore, we assume that the rate at which T-cells kill tumour cells is a linearly decreasing function of their exhaustion level, *j*, of the form:

$$ \hat{T}_{j} + \hat{N} \xrightarrow {\lambda \left( 1 - \frac{j}{n} \right) } \hat{T}_{j} + \hat{N}_{\text {dead}}, \qquad \qquad j = 0,1, \ldots , n, $$

where the parameter $\lambda$ denotes the tumour-cell kill rate for a fully active T-cell. We close the model by imposing the following initial conditions:

4 $$\begin{aligned} \hat{T}_0(0) =&\frac{\sigma _0}{k_0 n + \gamma }, \end{aligned}$$

5 $$\begin{aligned} \hat{T}_j(0) =&\frac{k_0(n-j+1)}{\gamma + k_0(n-j)} \hat{T}_{j-1}(0), \qquad j = 1,\ldots , n, \end{aligned}$$

6 $$\begin{aligned} \hat{N}(0) =&\hat{N}_0. \end{aligned}$$

In equations (4)–(5), we assume that initially there is a population of healthy T-cells resident in the tissue. We take this distribution to be the steady state distribution in the absence of tumour cells. We assume further that the tumour comprises $\hat{N}_0 > 0$ cells before a cytotoxic immune response is initiated.

### Reduced ODE Model

We now use equations (1)–(2) to derive a closed system of ODEs for the total number of T-cells, $\hat{\Theta }$, and their mean exhaustion level, $\mu$. Explicitly, we define:

$$ \hat{\Theta } = \sum _{j=0} ^{n} \hat{T}_{j} \qquad \text {and} \qquad \mu = \frac{1}{\hat{\Theta }} \sum _{j=0} ^{n} \frac{j}{n} \hat{T}_{j}. $$

Differentiating these expressions with respect to time $\hat{t}$, and substituting from equations (1)–(2), we obtain the following system of ODEs for $\hat{\Theta }$, $\mu$ and $\hat{N}$:

7 $$\begin{aligned} \frac{d\hat{\Theta }}{d\hat{t}}&= \sigma _{0} + \frac{\sigma _{1}\hat{N}}{1 + \left( \frac{\hat{N}}{\bar{N}} \right) ^2} - \gamma \hat{\Theta }, \end{aligned}$$

8 $$\begin{aligned} \frac{d\mu }{d\hat{t}}&= (k_{0} + k_{1}\hat{N})(1 - \mu ) - \frac{\mu }{\hat{\Theta }} \left( \sigma _{0} + \frac{\sigma _{1}\hat{N}}{1 + \left( \frac{\hat{N}}{\bar{N}} \right) ^2} \right) , \end{aligned}$$

9 $$\begin{aligned} \frac{d\hat{N}}{d\hat{t}}&= r\hat{N} \left( 1 - \frac{\hat{N}}{K} \right) - \lambda \hat{N} \hat{\Theta } (1-\mu ), \end{aligned}$$

with initial conditions:

10 $$\begin{aligned} \hat{\Theta }(0) = \frac{\sigma _0}{\gamma }, \qquad \mu (0) = \frac{k_0}{k_0 + \gamma }, \qquad \hat{N}(0) = \hat{N}_0. \end{aligned}$$

We will refer to equations (7)–(9) as the **reduced ODE model**. Equation (7) reflects our assumptions that T-cells infiltrate from the bloodstream at a baseline rate $\sigma _0$ and are additionally recruited at a rate $\frac{\sigma _{1}\hat{N}}{1 + \left( \frac{\hat{N}}{\bar{N}} \right) ^2}$ due to the tumour cells. Equation (8) shows how the mean exhaustion level of T-cells increases with the number of tumour cells and decreases as T-cell recruitment increases. This reflects our assumption that T-cells are fully active when they infiltrate the tumour region, and so reduce the mean exhaustion level.

Since there is no explicit dependence on $\{ \hat{T}_j: j = 0,1,\ldots ,n \}$, the reduced ODE model decouples from the exhaustion-structured model of T-cells. In particular, equations (7)–(10) define a closed initial value problem for $\hat{\Theta }, \mu$ and $\hat{N}$, that can be studied independently of equations (1)–(2). We remark that we can further derive an expression for the variance of the T-cell exhaustion distribution (see Appendix A.1 for more details). Similar reduction of a structured mathematical model to a closed system of ODEs has been seen in models of lipid accumulation in macrophages in atherosclerosis (Chambers et al. 2023).

### Non-dimensionalisation

We make the following scaling choices to non-dimensionalise the model:

$$ \hat{t} = \frac{1}{r} t, \qquad \hat{N} = KN, \qquad \hat{\Theta } = \frac{\sigma _{0}}{r} \Theta . $$

Since we are interested in the effects of T-cell exhaustion on the tumour’s growth dynamics, we scale time with the tumour growth rate, *r*. We scale the tumour cell population, $\hat{N}$, with its carrying capacity, *K*, and note that approaching this capacity signifies ‘tumour escape’ as per the 3 Es of immunoediting (Dunn et al. 2004). Lastly, we scale the total number of T-cells, $\hat{\Theta }$, with the basal influx that occurs during the time scale, $r^{-1}$, associated with tumour growth. With these scalings (and noting that $\mu \in [0,1]$ is, by definition, dimensionless), the reduced ODE model becomes:

11 $$\begin{aligned} \frac{d\Theta }{dt}&= 1 + \frac{ \tilde{\sigma } N}{1 + \left( \frac{N}{\tilde{N}} \right) ^2} - \tilde{\gamma } \Theta , \end{aligned}$$

12 $$\begin{aligned} \frac{d\mu }{dt}&= ( \kappa _0 + \kappa _1 N )(1 - \mu ) - \frac{\mu }{\Theta } \left( 1 + \frac{ \tilde{\sigma } N}{1 + \left( \frac{N}{\tilde{N}} \right) ^2} \right) , \end{aligned}$$

13 $$\begin{aligned} \frac{dN}{dt}&= N( 1 - N) - \tilde{\lambda } N \Theta (1-\mu ), \end{aligned}$$

with initial conditions,

14 $$\begin{aligned} \Theta (0) = \frac{1}{\tilde{\gamma }}, \qquad \mu (0) = \frac{\kappa _0}{\kappa _0 + \tilde{\gamma }}, \qquad N(0) = N_0, \end{aligned}$$

where we have introduced the following dimensionless parameter groupings:

$$ \tilde{\sigma } = \frac{\sigma _{1} K}{\sigma _{0}}, \qquad \kappa _0 = \frac{k_0}{r}, \qquad \kappa _1 = \frac{k_1 K}{r}, \qquad \tilde{\gamma } = \frac{\gamma }{r}, \qquad \tilde{\lambda } = \frac{\lambda \sigma _0}{r^2}, \qquad \tilde{N} = \frac{\bar{N}}{K}, \qquad N_0 = \frac{\hat{N}_0}{K}. $$

Similarly, the exhaustion-structured ODEs for the T-cell population become,

15 $$\begin{aligned} \frac{dT_{0}}{dt}&= 1 + \frac{ \tilde{\sigma } N}{1 + \left( \frac{N}{\tilde{N}} \right) ^2} - ( \kappa _0 + \kappa _1 N ) nT_{0} - \tilde{\gamma } T_{0}, \end{aligned}$$

16 $$\begin{aligned} \frac{dT_{j}}{dt}&= ( \kappa _0 + \kappa _1 N )n \left[ \left( 1 - \frac{j-1}{n} \right) T_{j-1} - \left( 1 - \frac{j}{n} \right) T_{j} \right] - \tilde{\gamma } T_{j}, \qquad j = 1,2, \ldots ,n, \end{aligned}$$

with initial conditions,

17 $$\begin{aligned} T_0(0) = \frac{1}{\kappa _0 n + \tilde{\gamma }} \quad \text {and} \quad T_j(0) = \frac{\kappa _0(n-j+1)}{\tilde{\gamma } + \kappa _0(n-j)} T_{j-1}(0), \qquad j = 1,\ldots , n. \end{aligned}$$

### Parameter Values

**Table 1:** Ranges of values for dimensional model parameters

Parameter	Value (range)	Description	Source
*r*	0.18 day$^{-1}$	tumour growth rate	Kuznetsov et al. (1994),
	(0.1–0.5 day$^{-1}$)		López et al. (2014),
			Wilkie and Hahnfeldt (2013)
*K*	5$\times$10$^{8}$ cells	tumour carrying capacity	Kuznetsov et al. (1994),
	(10$^8$–10$^{10}$ cells)		López et al. (2014),
			Wilkie and Hahnfeldt (2013),
			Kirschner and Panetta (1998)
$\lambda$	1.101$\times$10$^{-7}$ day$^{-1}$ cells$^{-1}$	rate of T-cell induced tumour	Kuznetsov et al. (1994),
	(10$^{-8}$–10$^{-4}$ day$^{-1}$ cells$^{-1}$)	kill, per T-cell	Liao et al. (2014),
			Wilkie and Hahnfeldt (2013)
$\sigma _0$	1.3$\times$10$^4$ cells day$^{-1}$	basal rate of T-cell influx	Kuznetsov et al. (1994)
$\sigma _1$	0.049 day$^{-1}$	2$\times$ max rate of tumour-stimulated	Dritschel et al. (2018),
	(0.001–0.05 day$^{-1}$)	influx of T-cells, per tumour cell	Kirschner and Panetta (1998),
			Kuznetsov et al. (1994)
$\bar{N}$	2$\times$10$^7$ cells	tumour cell number at which T-cell	Kuznetsov et al. (1994),
		influx is maximal	
$k_0$	0.01 day$^{-1}$	basal rate of T-cell exhaustion	estimated
	(0.001–0.01 day$^{-1}$)		
$k_1$	5$\times$10$^{-10}$ cells$^{-1}$ day$^{-1}$	enhanced rate of exhaustion	estimated
	(10$^{-11}$–10$^{-8}$ cells$^{-1}$ day$^{-1}$)	per tumour cell	
$\gamma$	0.0412 day$^{-1}$	natural death rate of T-cells	Kuznetsov et al. (1994),
	(0.02–0.05 day$^{-1}$)		Liao et al. (2014),
			Pillis et al. (2005)
$\hat{N}_0$	10$^7$ cells	initial number of tumour cells	estimated
	(10$^{7}$–10$^{8}$ cells)		

In Table 1, we summarise the range of parameter values reported in biological studies and the mathematical modelling literature, and present the specific values that we use for the remainder of the paper. Since there is a lack of experimental data available investigating T-cell exhaustion, we propose estimates for exhaustion parameters. We anticipate that T-cell exhaustion will occur on a timescale that is similar, but slightly longer, than that for natural cell death, so we assume $0< k_1 < \gamma$. On exposure to chronic antigen stimulation, CD8+ T-cells are thought to become exhausted over a period of days or weeks (Blank et al. 2019). Therefore, we assume $k_1 \hat{N}(\hat{t}) \sim \frac{1}{14}$, and estimate that the exhaustion parameter will lie in the range $\frac{1}{14 K}< k_1 < \frac{1}{14 \hat{N}_0}$.

In Table 2, we state the dimensionless parameter values that correspond to the chosen values of the dimensional parameters. For consistency, most parameter values are taken from Kuznetsov et al. (1994), and were estimated by comparing a mathematical model for immunogenic tumour growth with experimental data from the spleen of mice. We use the parameter values in Table 2 throughout the paper, unless stated otherwise. We emphasise that our choices of parameter values are representative, and that our model is intended to generate qualitative understanding of the impact of exhaustion on tumour growth dynamics.

**Table 2:** Values of dimensionless model parameters used in the remainder of the paper

Parameter	Value	Description
(dimensionless)		
$\tilde{\lambda }$	0.0442	Rate of T-cell induced tumour death per T-cell
$\tilde{\sigma }$	1885	Rate of T-cell influx
$\tilde{N}$	0.04	Value of *N*(*t*) at which T-cell influx is maximal
$\kappa _0$	0.0556	Basal rate of T-cell exhaustion
$\kappa _1$	1.39	Enhanced rate of T-cell exhaustion per tumour cell
$\tilde{\gamma }$	0.229	Natural death rate of T-cells

## Results

In this section, we analyse the behaviour of the reduced ODE model (11)–(13) and the corresponding exhaustion dynamics of the T-cell population (15)–(16). We begin by presenting numerical simulations that illustrate the different qualitative behaviours that the model exhibits. In so doing, we show that the model recapitulates the 3 E’s of immunoediting (Dunn et al. 2004) and indicate how different T-cell exhaustion profiles are associated with different tumour outcomes. We then consider the system’s long-term behaviour by identifying its steady states and characterising their linear stability. Using these results, we perform bifurcation analysis to identify distinct parameter regions based on the number of steady states that the model exhibits, their qualitative behaviour and stability. Finally, we incorporate treatment into the model and present numerical solutions that illustrate its impact on model dynamics.

### Model Dynamics

**Fig. 2:**
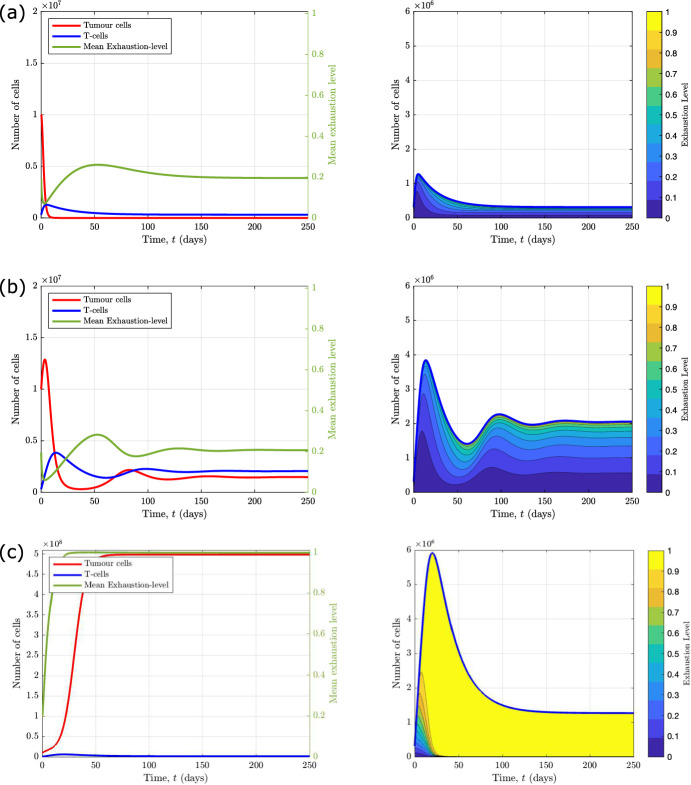
Numerical simulations illustrating the different qualitative behaviours that the model exhibits for different values of $\tilde{\lambda }$ and $\kappa _1$. On the left, we present solutions to equations (11)–(13) that show the evolution of the number of tumour cells (red), the total number of T-cells (blue), and their mean exhaustion level (green) over a period of 250 days. On the right, we present solutions to equations (15)–(16) that show the evolution of the corresponding T-cell exhaustion distribution. We re-dimensionalise the solutions to show the relative population cell counts. We choose three sets of parameter values for the rate of T-cell induced tumour kill, $\tilde{\lambda }$, and the exhaustion rate per tumour cell, $\kappa _1$: **a** when $\tilde{\lambda } = 0.29$ and $\kappa _1 = 1.39$ we observe tumour elimination; **b** when $\tilde{\lambda } = 0.0442$ and $\kappa _1 = 1.39$ we observe tumour equilibrium; **c** when $\tilde{\lambda } = 0.0442$ and $\kappa _1 = 80.0$ we observe tumour escape. The remaining parameters are fixed at the default values stated in Table 2, with $n=10$ (Color figure online)

In Fig. 2, we present numerical solutions of equations (11)–(13) which illustrate the different qualitative behaviours that the model exhibits as $\tilde{\lambda }$ and $\kappa _1$ vary. Increasing these parameters corresponds to increasing the rates of T-cell induced tumour death and T-cell exhaustion respectively. We also present the corresponding numerical solutions of equations (15)–(16) which show how the T-cell exhaustion distribution evolves over time in each case. We solve the ODEs using MATLAB’s ode45 solver, which is based on an explicit Runge–Kutta (4,5) formula, the Dormand-Prince pair.

In Fig. 2a, we observe tumour elimination because we have an effective immune response. The number of T-cells spikes initially due to the presence of the tumour which increases the rate at which T-cells are recruited to the TME. The T-cells outcompete the tumour cells, killing them at a rate which exceeds the rate of tumour cell proliferation, leading to tumour elimination. Furthermore, the T-cells eliminate the tumour so rapidly that they do not experience high levels of exhaustion. At long times, the system reverts to a tumour-free steady state, for which the exhaustion distribution of the T-cells is monotonically decreasing (i.e., skewed towards active T-cells).

The results presented in Fig. 2b show how the system dynamics change when the rate at which T-cells kill tumour cells is reduced (from $\tilde{\lambda } = 0.29$ to $\tilde{\lambda } = 0.0442$). In this case, the initial rate of tumour cell proliferation exceeds the rate of T-cell induced cell death. However, the rapid rise in tumour cell numbers increases the rate at which T-cells are recruited to the TME and, hence, the total rate of T-cell induced tumour cell death. As the tumour shrinks, T-cell recruitment also decreases. As T-cell numbers fall, the rate at which they kill tumour cells also falls, and tumour cell numbers start to increase again. This predator–prey like behaviour continues, with damped oscillations, until the system settles to an equilibrium where the tumour cells and T-cells co-exist. Comparison with Fig. 2a reveals that, in this case, the equilibrium T-cell population is larger and more uniformly distributed across the exhaustion spectrum. We note that for these parameter values, the system is bistable; for a larger initial tumour-cell population, the system evolves to tumour escape (see Appendix A.2).

In Fig. 2c, we show how the system dynamics change when the rate of T-cell exhaustion per tumour cell is increased (from $\kappa _1 = 1.39$ to $\kappa _1 = 80.0$). As in Fig. 2a and b, the tumour grows initially, causing a large increase in the influx of T-cells into the TME. However, the T-cells quickly become exhausted, which reduces their cytotoxic activity so that the rate of tumour cell proliferation greatly exceeds the rate of T-cell induced tumour cell death. The tumour cells then increase in number, with little opposition from the T-cells, until they reach their carrying capacity—we term this behaviour ‘tumour escape’. At long times, the T-cell population reaches a large but chronically exhausted steady state, characterised by a distribution which is monotonically increasing with exhaustion levels (i.e., skewed towards fully exhausted T-cells). Notice that the number of tumour cells for tumour escape is much larger than for tumour elimination or equilibrium (compare Fig. 2c with Fig. 2a and b).

Figure 2 shows how the distribution of T-cells across exhaustion states evolves during a tumour’s evolution, and suggests how different exhaustion distributions may be associated with different tumour outcomes—in particular, whether the tumour escapes, attains an equilibrium, or is eliminated by the T-cells. These results suggest that analysing the exhaustion distribution of T-cells from patient samples could be used to predict whether a tumour will be eliminated or contained by the immune system or escape immune control.

### Steady State Analysis

Let $\Theta ^*, \mu ^*, N^*$ denote the steady state solutions for the total number of T-cells, their mean exhaustion value, and the total number of tumour cells, respectively. By setting $d/dt = 0$ in equations (11)–(13), we derive the following expressions for $\Theta ^*$ and $\mu ^*$ in terms of $N^*$:

18 $$\begin{aligned} \Theta ^* = \frac{1}{\tilde{\gamma }} \left( 1 + \frac{\tilde{\sigma } N^*}{1 + \left( \frac{N^*}{\tilde{N}} \right) ^2} \right) \quad \text {and} \quad \mu ^* = \frac{\kappa _0 + \kappa _1 N^*}{\tilde{\gamma } + \kappa _0 + \kappa _1 N^*}. \end{aligned}$$

In equation (18), $N^*=0$ or $N^* > 0$ solves the polynomial,

19 $$\begin{aligned} 1 - N^* = f(N^*) \quad \text{ where } \quad f(N^*) :=\frac{ \tilde{\lambda }}{\tilde{\gamma } + \kappa _0 + \kappa _1 N^*} \left( 1 + \frac{\tilde{\sigma } N^*}{1 + \left( \frac{N^*}{\tilde{N}} \right) ^2} \right) . \end{aligned}$$

By considering intersections of the graph of $f(N^*)$ with the line $1 - N^*$, we deduce that the number of solutions to equation (19) depends on the value of $f(0) = \frac{\tilde{\lambda }}{\tilde{\gamma } + \kappa _0}$, and we consider the following two cases:

1. If $\frac{\tilde{\lambda }}{\tilde{\gamma } + \kappa _0} < 1$, then equation (19) admits one or three solutions;
2. If $\frac{\tilde{\lambda }}{\tilde{\gamma } + \kappa _0} > 1$, then equation (19) admits zero, two or four solutions.

**Fig. 3:**
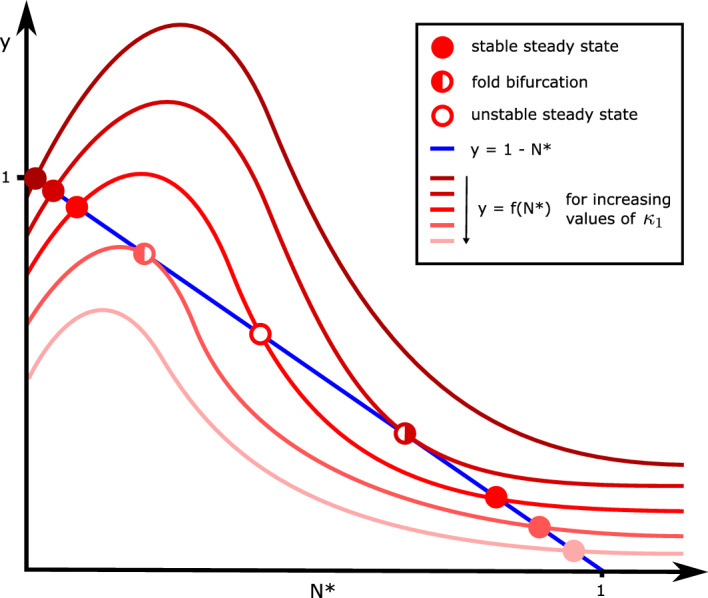
Schematic showing how intersections between the curves $y = f(N^*)$ and $y = 1 - N^*$ and, hence, the number of non-trivial solutions to equation (19) change as $\kappa _1$ varies. We sketch the curve $y = f(N^*)$ (red) for 5 different values of $\kappa _1$ (the rate of T-cell exhaustion per tumour cell), to illustrate the different qualitative ways in which it intersects with the line $y = 1 - N^*$ (blue). The points of intersection are indicated by circles: filled circles represent stable steady state solutions; unfilled circles represent unstable steady state solutions; half-filled circles represent fold bifurcations, where a stable and unstable steady state coalesce/emerge. We assume $f(0) = \frac{\tilde{\lambda }}{\tilde{\gamma } + \kappa _0} < 1$, in which case equation (19) has either one or three non-trivial solutions (Color figure online)

In Fig. 3, we present a graphical representation of the solutions to equation (19) for $\frac{\tilde{\lambda }}{\tilde{\gamma } + \kappa _0} < 1$ (see Appendix A.2 for an analogous schematic for the case $\frac{\tilde{\lambda }}{\tilde{\gamma } + \kappa _0} > 1$). We remark that in the special case, $\frac{\tilde{\lambda }}{\tilde{\gamma } + \kappa _0} = 1$, equation (19) admits between one and four solutions. We conclude that the reduced ODE model (11)–(13) possesses at most 5 steady state solutions.

In Sect. 3.4, we use (18) and (19) to show how the steady state solutions of the reduced ODE model change as we vary key model parameters. Using the range of parameter values presented in Sect. 2.4, we find that there are at most four physically realistic steady states solutions. For completeness, in Sect. 3.5, we demonstrate the existence of parameter values for which the system exhibits the maximum number of five physically realistic steady state solutions and characterise their stability.

For completeness, before presenting the steady state solutions of the reduced ODE model, we first derive expressions for the corresponding steady-state T-cell exhaustion distribution. Setting $d/dt=0$ in equations (15) and (16), it is straightforward to obtain the following expressions for $T_0^*$ and $T_j^* \; (j = 1, \ldots , n$):

20 $$\begin{aligned} T_0^*&= \frac{1}{(n+ 1 - p^*) (\kappa _0 + \kappa _1 N^*) } \, \left( 1 + \frac{ \tilde{\sigma } N^*}{1 + \left( \frac{N^*}{\tilde{N}} \right) ^2} \right) , \nonumber \\ T_j^*&= \frac{(n-j+1)}{(n-j+1-p^*)} \, T_{j-1}^*, \qquad \text{ for } \; j = 1,\ldots , n, \end{aligned}$$

where we define $p^* = p(N^*) :=1 - \frac{\tilde{\gamma }}{(\kappa _0 + \kappa _1 N^*)}$ as a function of the number of tumour cells at steady state, $N^*$, with $p^* \in \left[ 1 - \frac{\tilde{\gamma }}{\kappa _0}, 1 \right)$.

We note that the value of $p^* = p(N^*)$ characterises the skew of the steady state exhaustion distribution of the T-cell population:

- if $N^* > \frac{\tilde{\gamma } - \kappa _0}{\kappa _1}$, then $p(N^*) > 0$ and the T-cell exhaustion distribution is convex increasing, which corresponds to the T-cells accumulating at high levels of exhaustion
- for $N^* < \frac{\tilde{\gamma } - \kappa _0}{\kappa _1}$, $p(N^*) < 0$ and the T-cell exhaustion distribution is monotonically decreasing and, therefore, skewed towards active T-cells
- for the special case when $N^* = \frac{\tilde{\gamma } - \kappa _0}{\kappa _1}$, $p(N^*) = 0$ and the T-cell population is uniformly distributed across exhaustion levels

We also note that the exhaustion parameters, $\kappa _0$ and $\kappa _1$, and the rate of T-cell clearance, $\tilde{\gamma }$, determine the qualitative exhaustion distribution of the T-cell population at steady state. In particular, while the influx parameters $\tilde{\sigma }$ and $\tilde{N}$ control the number of active T-cells that infiltrate the TME, they do not affect the skewedness at steady state.

### Linear Stability Analysis

We characterise the linear stability of the steady state solutions, to determine which steady state will be attained at long times. It is straightforward (see Appendix A.3 for details) to show that the eigenvalues, $\alpha$, of the linearised system satisfy:

21 $$\begin{aligned} 0&= - (\alpha + \tilde{\gamma }) \Bigl [ (\alpha + \tilde{\gamma } + k(N^*) ) \left( \alpha + f(N^*) - (1-2N^*) \right) \nonumber \\&\quad - N^* \Big ( f(N^*) k'(N^*) - \tilde{\lambda }\sigma '(N^*) \Big ) \Bigr ] , \end{aligned}$$

where the functions $k(N) = \kappa _0 + \kappa _1 N$ and $\sigma (N) = 1 + \frac{ \tilde{\sigma } N}{1 + \left( \frac{N}{\tilde{N}} \right) ^2}$ represent the dimensionless exhaustion rate and T-cell influx rate, and where *f*(*N*) is defined in equation (19). We use $'$ to denote differentiation with respect to *N*. For a stable steady state, we require Re($\alpha ) < 0$ for all eigenvalues, $\alpha$.

For the tumour-free steady state, $N^* = 0$, we have:

$$ \alpha _1 = - \tilde{\gamma }, \quad \alpha _2 = - (\tilde{\gamma } + \kappa _0), \quad \alpha _3 = 1 - f(0) = 1 - \frac{\tilde{\lambda }}{\tilde{\gamma } + \kappa _0}. $$

Since $\tilde{\gamma } > 0$ and $\kappa _0 > 0$, we deduce that $N^* = 0$ is unstable if $\frac{\tilde{\lambda }}{\tilde{\gamma } + \kappa _0} < 1$, and linearly stable otherwise. We conclude that a necessary condition for tumour elimination to occur is $\tilde{\lambda } > \tilde{\gamma } + \kappa _0 :=\lambda _{\text {tr}}$. We note also that when $\tilde{\lambda } = \lambda _{\text {tr}}$, the trivial solution $N^* = 0$ is a degenerate steady state solution. Based on these observations, we anticipate that when $\tilde{\lambda } = \lambda _{\text {tr}}$ there is a transcritical bifurcation at which the trivial steady state intersects and exchanges stability with a second steady state solution.

For physically realistic, non-trivial steady state solutions, $N^* > 0$ and $f(N^*) = 1 - N^*$ (19). Substituting from equation (19) into equation (21), we have that,

22 $$\begin{aligned} - (\alpha + \tilde{\gamma }) \Bigl [ \alpha ^2 + (\tilde{\gamma } + k(N^*) + N^*) \alpha - N^* g(N^*) \Bigr ] = 0, \end{aligned}$$

where the function $g(N^*)$ is defined as follows:

23 $$\begin{aligned} g(N^*) :=- \tilde{\gamma } - k(N^*) + ( 1 - N^*) k'(N^*) - \tilde{\lambda }\sigma '(N^*). \end{aligned}$$

Since $(\tilde{\gamma } + k(N^*) +N^*) > 0$, we deduce that the non-trivial steady state is unstable if $g(N^*) > 0$, and linearly stable if $g(N^*) < 0$ (and, similarly, for the stability of the steady state T-cell distribution; see Appendix A.3 for more details). We numerically compute the value of $g(N^*)$ for each steady state to determine its linear stability. We set $g(N^*) = 0$ to find fold points at which the linear stability of a steady state switches.

### Bifurcation Analysis

We now combine the analytical results from Sects. 3.2 and 3.3 to show how the number of steady state solutions and their linear stability change as we vary the rate of T-cell induced tumour kill ($\tilde{\lambda }$) and the rate at which tumour cells accelerate T-cell exhaustion ($\kappa _1$). In so doing, we focus on parameters relating to T-cell activity that may vary between patients and/or that are potential targets for immunotherapies. For example, in CAR T-cell therapy T-cells are removed from the blood and genetically engineered to more efficiently locate and kill tumour cells (Raskov et al. 2021). Alternatively, immune checkpoint inhibitors block negative immunoregulatory pathways, such as PD-1 and CTLA-4, that are characteristic of T-cell exhaustion (Waldman et al. 2020). In our model, these treatments would induce an increase in $\tilde{\lambda }$ and a decrease in $\kappa _1$, respectively.

As shown in Sect. 3.2, the model admits a tumour-free steady state, which can be stable or unstable, and up to four additional, physically-realistic steady state solutions, all characterised by a positive tumour burden and at most two of which are stable. Therefore, there are at most three stable tumour-steady-state branches. We define model solutions that evolve to the larger non-zero tumour-steady-state branch as tumour escape, those that evolve to the smaller, non-zero tumour-steady-state branch as tumour equilibrium, and those that evolve to the tumour-free steady-state branch as tumour elimination.

**Fig. 4:**
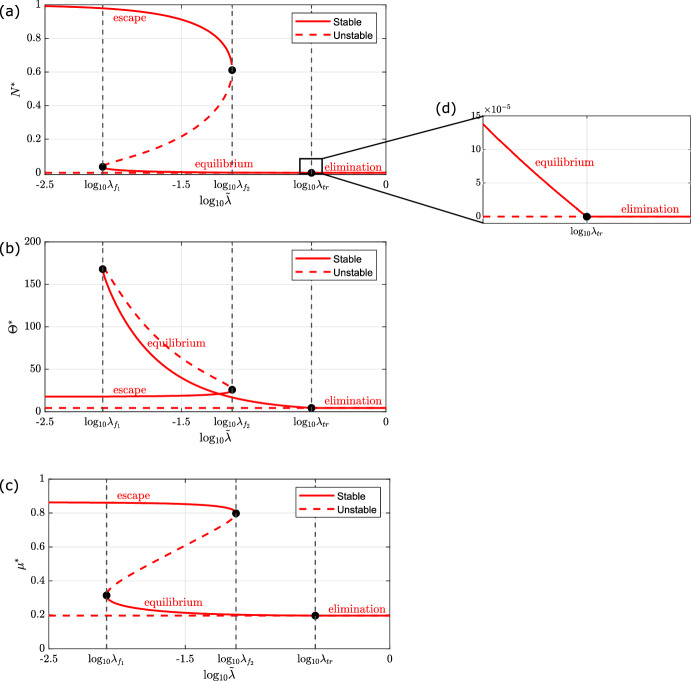
Series of bifurcation diagrams showing how the existence and stability of the steady state solutions of equations (11)–(13) change as $\tilde{\lambda }$ varies. We present the steady states for **a** the number of tumour cells ($N^*$), **b** the number of T-cells ($\Theta ^*$), and **c** the mean exhaustion level ($\mu ^*$) as we vary $\tilde{\lambda }$, the rate of T-cell induced tumour kill. Stable and unstable steady states are denoted with solid and dashed lines respectively. Parameter space is partitioned by vertical black dashed lines which pass through two fold points, at $\tilde{\lambda } = \lambda _{f_1}, \lambda _{f_2}$, and a transcritical bifurcation, at $\tilde{\lambda } = \lambda _{\text {tr}} :=\tilde{\gamma } + \kappa _0$ (see inset in **d**). The system is bistable for $\lambda _{f_1}< \tilde{\lambda } < \lambda _{f_2}$, and monostable otherwise. There are three distinct branches of stable steady state solutions, corresponding to ‘tumour escape’ (the upper branch in **a**), ‘tumour equilibrium’ (the lower, non-trivial branch in **a**), and ‘tumour elimination’ (the trivial, tumour-free branch in **a**). Except for $\tilde{\lambda }$, all parameters are fixed at the default values listed in Table 2 (Color figure online)

We begin by focusing on $\tilde{\lambda }$ as a bifurcation parameter. The results are presented in Fig. 4 and can be summarised as follows:

- $0< \tilde{\lambda } < \lambda _{f_1}:$ The system is monostable. For all physically realistic initial conditions, it evolves to a unique stable steady state, corresponding to ‘tumour escape’, characterised by a large tumour burden, near its carrying capacity, and a small number of T-cells exhibiting high levels of exhaustion.
- $\tilde{\lambda } = \lambda _{f_1}:$ There is a fold bifurcation at which a steady state solution characterised by a small number of tumour cells and a large number of active T-cells is created.
- $\lambda _{f_1}< \tilde{\lambda } < \lambda _{f_2}:$ The system is bistable. Depending on the initial numbers of tumour cells and T-cells, the system evolves to either a stable steady state corresponding to ‘tumour escape’ (i.e., a large tumour burden and a small number of highly exhausted T-cells) or ‘tumour equilibrium’ (i.e., a smaller tumour burden which decreases as $\tilde{\lambda }$ increases, and a large T-cell population whose size and mean exhaustion level decrease as $\tilde{\lambda }$ increases).
- $\tilde{\lambda } = \lambda _{f_2}:$ The ‘escape’ branch of stable steady state solutions collides with the intermediate branch of unstable steady state solutions at a saddle-node bifurcation.
- $\lambda _{f_1}< \tilde{\lambda } < \lambda _{\text {tr}}:$ The system is monostable. For all physically realistic initial conditions, it evolves to a unique stable steady state, corresponding to ‘tumour equilibrium’, characterised by a small tumour burden and a small number of T-cells with a low mean exhaustion level.
- $\tilde{\lambda } = \lambda _{\text {tr}}:$ The system undergoes a transcritical bifurcation when the stable branch of equilibrium solutions collides with, and exchanges stability with, the trivial branch of steady state solutions corresponding to ‘tumour elimination’ ($N^* = 0$).
- $\lambda _{\text {tr}} < \tilde{\lambda }:$ The system is monostable. For all physically realistic initial conditions, it evolves to a unique stable steady state, which corresponds to ‘tumour elimination’ ($N^* = 0$). The immune response is able to clear the tumour and a small number of circulating T-cells remain, with minimal mean exhaustion level.

**Fig. 5:**
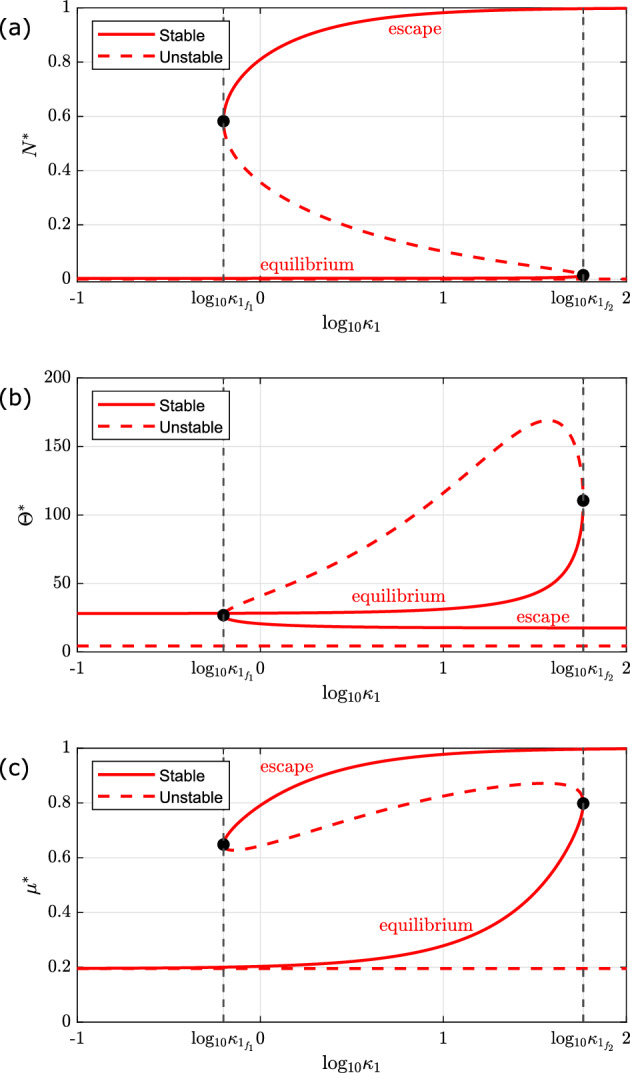
Series of bifurcation diagrams showing how the existence and stability of the steady state solutions of equations (11)–(13) change as $\kappa _1$ varies, for unstable tumour elimination. We present the steady state solutions for **a** the number of tumour cells ($N^*$), **b** the number of T-cells ($\Theta ^*$), and **c** the mean exhaustion level ($\mu ^*$) as we vary $\kappa _1$, the enhanced rate of T-cell exhaustion per tumour cell. Stable and unstable steady states are denoted with solid and dashed lines respectively. Parameter space is partitioned by vertical black dashed lines which pass through two fold points, at $\kappa _1 = \kappa _{1_{f_1}}, \kappa _{1_{f_2}}$. The system is bistable for $\kappa _{1_{f_1}}< \kappa _1 < \kappa _{1_{f_2}}$ and monostable otherwise. There are two distinct branches of stable steady state solutions, corresponding to ‘tumour escape’ (the upper branch in **a**), and ‘tumour equilibrium’ (the lower, non-trivial branch in **a**). Except for $\kappa _1$, all parameters are fixed at the default values listed in Table 2. For these parameter values, ‘tumour elimination’ is unstable (Color Figure Online)

**Fig. 6:**
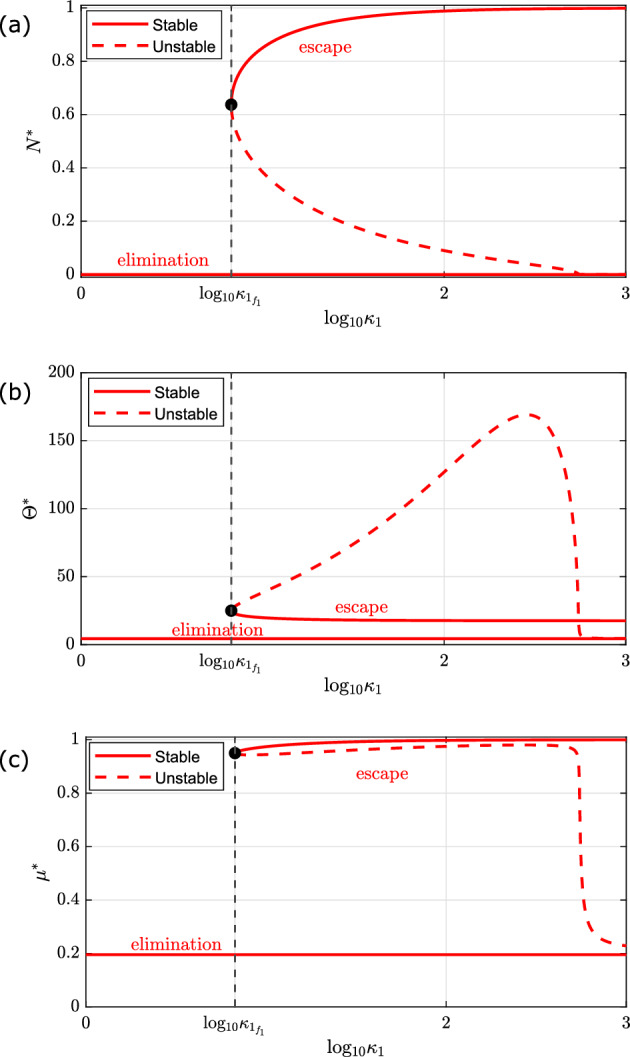
Series of bifurcation diagrams showing how the existence and stability of the steady state solutions of equations (11)–(13) change as $\kappa _1$ varies, for stable tumour elimination. We present the steady state solutions for **a** the number of tumour cells ($N^*$), **b** the number of T-cells ($\Theta ^*$), and **c** the mean exhaustion level ($\mu ^*$) as we vary $\kappa _1$, the enhanced rate of T-cell exhaustion per tumour cell. Stable and unstable steady states are denoted with solid and dashed lines respectively. Parameter space is partitioned by vertical black dashed lines which pass through one fold point at $\kappa _1 = \kappa _{1_{f_1}}$. The system is bistable for $\kappa _{1_{f_1}} < \kappa _1$ and monostable otherwise. There are two distinct branches of stable steady state solutions, corresponding to ‘tumour escape’ (the upper branch in **a**), and ‘tumour elimination’ (the trivial, tumour-free branch in **a**). Except for $\kappa _1$ and $\tilde{\lambda } = 0.29$, all other parameters are fixed at the default values listed in Table 2. For these parameter values, ‘tumour elimination’ is stable (Color Figure Online)

Next we consider the role of T-cell exhaustion on the system’s long term dynamics by varying $\kappa _1$, the rate at which tumour cells increase the rate of T-cell exhaustion. We consider two cases, which differ in terms of the stability of the tumour-free steady state corresponding to ‘tumour elimination’, as determined by the value of $f(0) = \frac{\tilde{\lambda }}{\tilde{\gamma } + \kappa _0}$ (see Sect. 3.3). Our results are presented in Figs. 5 and 6, and can be summarised as follows.

**Case 1: Unstable Elimination** ($\frac{\tilde{\lambda }}{\tilde{\gamma } + \kappa _0} < 1$).

- $0< \kappa _1 < \kappa _{1_{f_1}}:$ The system is monostable. For all physically realistic initial conditions, it evolves to a unique stable steady state, corresponding to ‘tumour equilibrium’, characterised by a small tumour mass and a small number of T-cells with a low mean exhaustion level.
- $\kappa _1 = \kappa _{1_{f_1}}:$ There is a fold bifurcation at which a steady state solution characterised by a large number of tumour cells and a small number of largely exhausted T-cells is created.
- $\kappa _{1_{f_1}}< \kappa _1 < \kappa _{1_{f_2}}:$ In this region, the system is bistable. Depending on the initial numbers of tumour cells and T-cells, the system evolves to either a stable steady state corresponding to ‘tumour escape’ (i.e., a large tumour burden and a small number of highly exhausted T-cells) or ‘tumour equilibrium’ (i.e., a smaller tumour burden with a larger T-cell population whose size and mean exhaustion level increase as $\kappa _1$ increases).
- $\kappa _1 = \kappa _{1_{f_2}}:$ The ‘equilibrium’ branch of stable steady state solutions collides with the intermediate branch of unstable steady state solutions at a saddle-node bifurcation.
- $\kappa _{1_{f_2}} < \kappa _1:$ The system is monostable. For all physically realistic initial conditions, it evolves to a unique stable steady state, which corresponds to ‘tumour escape’, characterised by a large tumour burden, near its carrying capacity, and a small number of T-cells exhibiting high levels of exhaustion.

**Case 2: Stable Elimination** ($\frac{\tilde{\lambda }}{\tilde{\gamma } + \kappa _0} > 1$).

- $0< \kappa _1 < \kappa _{1_{f_1}}:$ The system is monostable. For all physically realistic initial conditions, it evolves to a unique stable steady state, corresponding to ‘tumour elimination’. The immune response is able to clear the tumour and a small number of circulating T-cells remain, with minimal mean exhaustion level.
- $\kappa _1 = \kappa _{1_{f_1}}:$ There is a fold bifurcation at which a steady state solution characterised by a large number of tumour cells and a small number of largely exhausted T-cells is created.
- $\kappa _{1_{f_1}} < \kappa _1:$ In this region, the system is bistable. Depending on the initial number of tumour cells and T-cells, the system evolves to either a stable steady state corresponding to ‘tumour escape’ (i.e., a large tumour burden and a small number of highly exhausted T-cells) or ‘tumour elimination’ (i.e., no tumour cells with a small circulating T-cell population with minimal exhaustion levels).

**Fig. 7:**
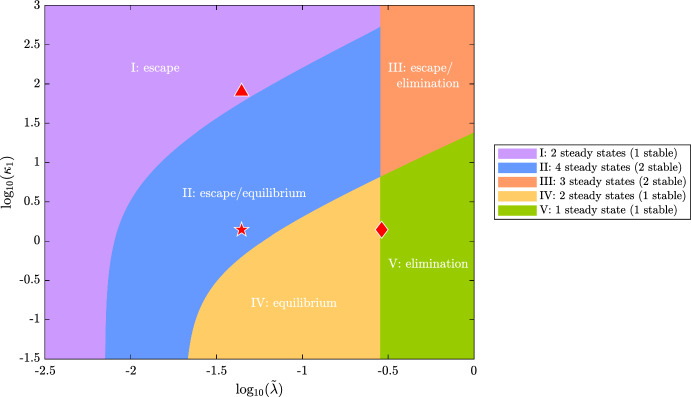
Bifurcation diagram showing how the number and nature of steady state solutions of equations (11)–(13) change as both $\tilde{\lambda }$ and $\kappa _1$ vary. We partition $(\tilde{\lambda }, \kappa _1)$ parameter space into 5 distinct regions according to the number of physically-relevant steady state solutions of the equations (11)–(13). Each region is numbered and labelled with the expected qualitative long-term behaviour for the parameter values in that region (i.e., the stable steady states), consistent with the 3 E’s of immunoediting (Dunn et al. 2004). The key (right) lists the number of physically-realistic steady state solutions and how many of which are stable. We identify the locations in parameter space of the parameter values used in the model simulations in Fig. 2: a red diamond where we see ‘tumour elimination’ in 2a ($\tilde{\lambda } = 0.29$, $\kappa _1 = 1.39$); a red star where we have bistability and observe ‘tumour equilibrium’ in 2b ($\tilde{\lambda } = 0.0442$, $\kappa _1 = 1.39$); a red triangle where we observe ‘tumour escape’ in 2c ($\tilde{\lambda } = 0.0442$, $\kappa _1 = 80$). Axes are plotted on a log scale. Except for $\tilde{\lambda }$ and $\kappa _1$, all parameters are fixed at the default values listed in Table 2 (Color figure online)

In Fig. 7, we show how the number, type, and stability of physically realistic steady state solutions change as $\tilde{\lambda }$ and $\kappa _1$ vary. We identify distinct regions of parameter space in which the model exhibits different qualitative behaviours. The curved boundaries track the locations of the fold points, on which $f(N^*) = 1 - N^*$ and $g(N^*) = 0$ (see equations (19) and (23)). The vertical boundary at $\tilde{\lambda } = \gamma + \kappa _0 = \lambda _{\text {tr}}$ delineates a transcritical bifurcation: as $\tilde{\lambda }$ increases across $\lambda _{\text {tr}}$, a stable ‘tumour equilibrium’ steady state ceases to be physically realistic and exchanges stability with the ‘tumour elimination’ steady state.

Figure 7 shows how variability in the strength and robustness of a patient’s immune response affects their tumour growth dynamics. In more detail, patients with small values of $\kappa _1$ and large values of $\tilde{\lambda }$ can eliminate their tumours, whereas those with large values of $\kappa _1$ and small values of $\tilde{\lambda }$ are unable to control them. Figure 7 also shows the importance of tailoring treatment to an individual, depending on their immune response. For example, patients who are characterised by large values of $\tilde{\lambda }$ and $\kappa _1$, are predicted to respond best to treatments that reduce the value of $\kappa _1$ such as immune checkpoint inhibitors (Waldman et al. 2020). By contrast, patients characterised by small values of $\tilde{\lambda }$ and $\kappa _1$, would respond best to treatment that increases the value of $\tilde{\lambda }$, such as CAR T-cell therapy (Raskov et al. 2021). We conclude that fitting our model to patient data could be used to identify which type of immunotherapy would be most beneficial for a particular patient.

### Identification of Tristability

**Fig. 8:**
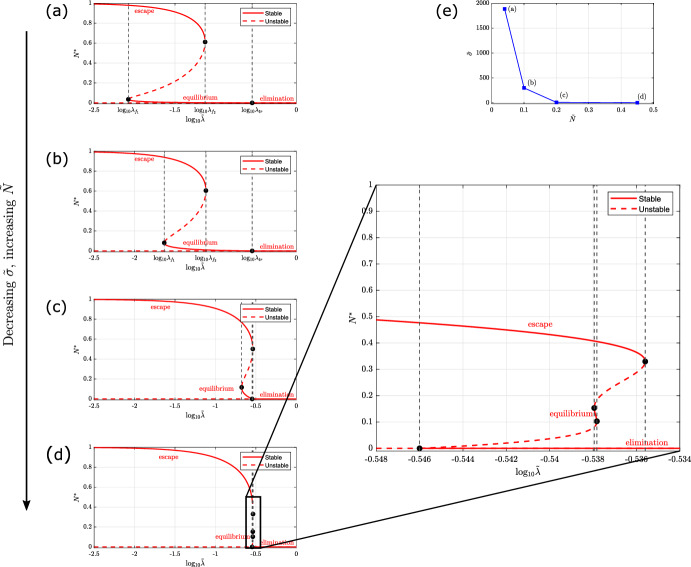
Series of bifurcation diagrams showing how the existence and stability of the tumour steady-state solutions of equations (11)–(13) change as $\tilde{\lambda }$ varies, progressively increasing the value of $\tilde{N}$ and decreasing the value of $\tilde{\sigma }$ to elicit 5 steady state solutions. We present a series of bifurcation diagrams, each showing the tumour steady state solutions of equations (11)–(13) and their linear stability as $\tilde{\lambda }$ varies. Stable and unstable steady states are denoted with red solid lines and red dashed lines respectively. Parameter space is partitioned by vertical black dashed lines which pass through fold points, $\tilde{\lambda } = \lambda _{f_i}$, and a transcritical bifurcation, $\tilde{\lambda } = \tilde{\gamma } + \kappa _0 = \lambda _{\text {tr}}$. Fold points in Figures **c** and **d** are ordered as following: **c** $\lambda _{f_1}< \lambda _{\text {tr}} < \lambda _{f_2}$; **d** $\lambda _{\text {tr}}< \lambda _{f_1}< \lambda _{f_2} < \lambda _{f_3}$. There are three distinct branches of stable steady state solutions, which correspond to ‘tumour escape’ (the upper stable branch), ‘tumour equilibrium’ (the lower, non-trivial stable branch), and ‘tumour elimination’ (the trivial tumour-free stable branch). We progressively increase the value of $\tilde{N}$ and decrease the value of $\tilde{\sigma }$ along a trajectory in parameter space, as shown in **e**, to elicit 5 steady state solutions: in **a**, $\tilde{\sigma } = 1885$ and $\tilde{N} = 0.04$; in **b**, $\tilde{\sigma } = 300$ and $\tilde{N} = 0.1$; in **c**, $\tilde{\sigma } = 10$ and $\tilde{N} = 0.2$; in **d**, $\tilde{\sigma } = 3.3$ and $\tilde{N} = 0.45$. Except for $\tilde{\lambda }$, $\tilde{\sigma }$, and $\tilde{N}$, all other parameters are fixed at the default values listed in Table 2. In **a**, the bifurcation diagram is the same as in Fig. 4a. In **d**, there is a range of parameters values of $\tilde{\lambda }$ where the system exhibits 5 steady state solutions, as indicated on the right (Color Figure Online)

In Sect. 3.4, we performed bifurcation analysis of the model based on parameter values retrieved from the literature, as detailed in Sect. 2.4. For these parameter values, we observe at most four physically realistic steady state solutions. However, our analysis in Sect. 3.2 revealed that the system may admit five physically realistic steady state solutions and so has the potential for tristability (3 simultaneously stable steady states). In this section, we identify a region of parameter space in which the model admits five steady state solutions and we characterise their linear stability. In more detail, we identify a trajectory in ($\tilde{N}, \tilde{\sigma }$) parameter space, as shown in Fig. 8e, along which the system transitions from admitting at most four to five physically realistic steady state solutions and characterise the system’s bifurcation structure at fixed points along this trajectory.

In Fig. 8, we show how the number of steady state solutions and their linear stability change as $\tilde{\lambda }$ varies, as we progressively decrease $\tilde{\sigma }$ and increase $\tilde{N}$. We recall that these parameters control the influx of T-cells into the TME: $\tilde{\sigma }$ corresponds to the initial linear rate of influx of active T-cells due to the tumour; $\tilde{N}$ corresponds to the number of tumour cells at which this influx is maximal. As we decrease $\tilde{\sigma }$ and increase $\tilde{N}$ along the trajectory shown in Fig. 8e, the fold points move closer together, and approach the transcritical bifurcation. Consequently, the parameter range in which we observe bistability becomes smaller. Ultimately, in Fig. 8d we identify a small range of parameter values, for which the model admits 5 physically-realistic steady state solutions. Furthermore, it is possible to show that, for these parameter values, the system is tristable and all 3 E’s of immunoediting (Dunn et al. 2004)—elimination, equilibrium, and escape—are simultaneously stable. In this parameter regime, the tumour can evolve to elimination, equilibrium, or escape depending on the initial conditions of the model.

### Implications for Treatment

Immune checkpoint inhibitors have been developed to combat T-cell exhaustion by blocking inhibitory receptors such as PD-1 and CTLA-4, and, thereby, reinvigorating the T-cells and enhancing the anti-tumour immune response (Budimir et al. 2022; Catakovic et al. 2017; Chow et al. 2022). We consider the impact on the system dynamics of a potential immunotherapy that transiently reduces $\kappa _1$, the rate of T-cell exhaustion per tumour cell. We model the effect of treatment by viewing $\kappa _1$ as a time-dependent parameter:

24 $$\begin{aligned} \kappa _1(t) = {\left\{ \begin{array}{ll} \kappa _1 & \text {for } 0 \le t< 100 \text { days,} \\ \kappa _1 - \Delta \kappa \frac{t - 100}{25} & \text {for } 100 \le t< 125 \text { days,}\\ \kappa _1 - \Delta \kappa & \text {for } 125 \le t< 175 \text { days,}\\ \kappa _1 - \Delta \kappa \frac{200 - t}{25} & \text {for } 175 \le t < 200 \text { days,}\\ \kappa _1 & \text {for } t \ge 200 \text { days.} \end{array}\right. } \end{aligned}$$

**Fig. 9:**
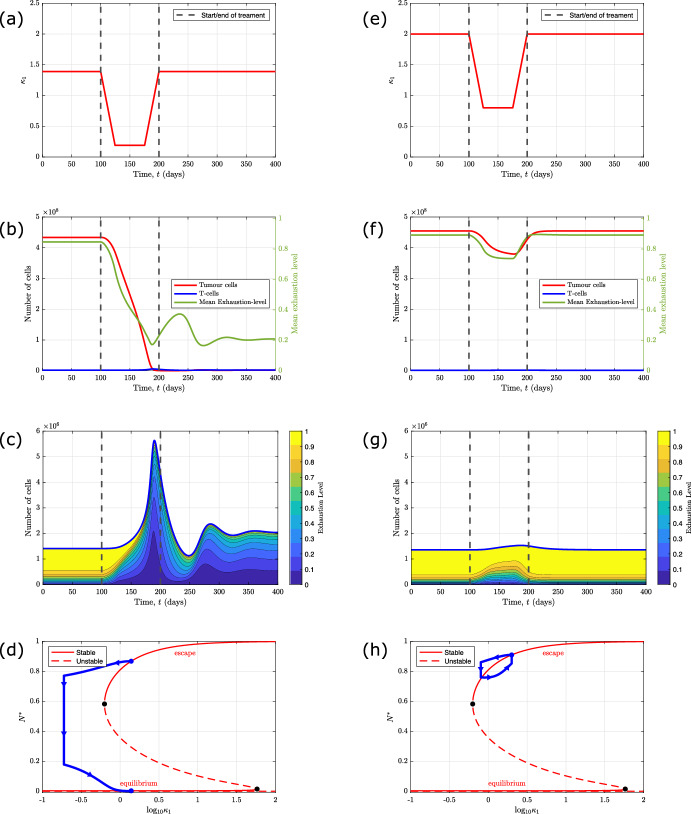
Numerical simulations reveal different qualitative responses to an immunotherapy which targets T-cell exhaustion. Panels **a** and **e** show how, for two different intrinsic values of $\kappa _1$, we model the effect of a single course of immunotherapy which causes a transient decrease in the rate of T-cell exhaustion per tumour cell, $\kappa _1$. When treatment starts (at $t=100$ days), the exhaustion rate decreases linearly from $\kappa _1$ to $\kappa _1 - \Delta \kappa$ over a period of 25 days. It is then held fixed for 50 days (from $t=125$ to $t = 175$ days), before it increases linearly to its original value at $t=200$ days. In panels **b** and **f**, we plot solutions to equations (11)–(13) which show how the number of tumour cells (red), the total number of T-cells (blue), and their mean exhaustion level (green) evolve in response to the treatment. In both panels, the initial conditions correspond to stable steady state solutions with a large tumour burden. In panels **c** and **g** we present solutions to equations (15)–(16) which show the evolution of the corresponding T-cell exhaustion distribution. In panels **d** and **h**, we present bifurcation diagrams for the tumour steady states as $\kappa _1$ varies, as in Fig. 5, and plot the trajectory of *N*(*t*), the number of tumour cells, as $\kappa _1$ varies during treatment. Panels **a**, **b**, **c**, and **d** show that, when $\kappa _1 = 1.39$, treatment causes a durable reduction in tumour burden. Panels **e**, **f**, **g**, and **h** show that when the intrinsic value of $\kappa _1$ is increased to $\kappa _1 = 2.0$, treatment elicits only a small and transient reduction in tumour burden. The remaining parameters are fixed at the default values stated in Table 2, with $n=10$ and $\Delta \kappa = 1.2$ (Color figure online)

In Fig. 9, we present simulation results for two different intrinsic values of $\kappa _1$, which show two different qualitative responses to treatment.

The results presented in Fig. 9a–d show that, when $\kappa _1 = 1.39$, treatment significantly reduces the tumour burden. At the start of treatment, the T-cell population becomes reinvigorated as the rate of exhaustion is reduced, and the T-cell exhaustion distribution shifts towards more active T-cells. The resulting increased cytotoxic activity significantly reduces the tumour burden, and further decreases the rate of exhaustion. At the end of treatment, the tumour population is small enough that the T-cells are able to contain the tumour’s growth even when the rate of exhaustion per tumour cell, $\kappa _1$, returns to its original value.

The results in Fig. 9e–h show that when the intrinsic value of $\kappa _1$ increases from $\kappa _1=1.39$ to $\kappa _1 = 2.0$, treatment has a minimal and transient effect on the tumour burden. At the start of treatment, the T-cell exhaustion distribution shifts to a more cytotoxic level (the mean exhaustion level decreases), but the effect is less pronounced than when $\kappa _1 = 1.39$. The increased cytotoxic activity of the T-cells produces only a modest reduction in the tumour burden. The tumour burden remains large throughout treatment, which limits the rate of infiltration of active T-cells so that the mean exhaustion-level remains high. At the end of treatment, when $\kappa _1$ returns to its original value, the T-cell exhaustion distribution shifts back towards the exhausted phenotype and the tumour population eventually returns to its original size.

Our model simulations explain why different patients may respond differently to the same treatment and highlight the importance of patient-specific treatment. The bifurcation diagrams in Fig. 9d and h explain why we observe different responses. In Fig. 9d (when $\kappa _1 = 1.39$), treatment enables the system to transition to the basin of attraction of the lower branch of stable steady state solutions, characterised by a small tumour burden. By contrast, in Fig. 9h (when $\kappa _1 = 2.0$), throughout treatment, the tumour remains in the basin of attraction of the upper solution branch, characterised by a large tumour burden. In future work, by fitting the model to patient data, the model could be used to identify patients who are likely to benefit from different types of immunotherapy.

## Equivalent PDE Model

In this section, we study a partial differential equation (PDE) model that describes how the exhaustion profile of the T-cell population changes over time. We derive this first-order, hyperbolic PDE by taking the limit as $n \rightarrow \infty$ of the discrete ODE model, defined by equations (15) and (16). We construct analytical solutions to the PDE model using the method of characteristics and compare them with numerical solutions of the discrete ODE model. In practice, T-cell exhaustion is often identified by measuring co-expression of multiple inhibitory receptors, such as PD-1 and CTLA-4, which are quantities that vary continuously and are not discrete. As such, the PDE model reflects our understanding of T-cell exhaustion as a continuous process and, in the longer term, provides a practical mathematical framework for validation against T-cell exhaustion data. The analytical solutions that it generates are more mathematically tractable and interpretable than those associated with the discrete ODE model and, as such, provide additional insight into T-cell exhaustion.

### Model Derivation

Let *T*(*s*, *t*) be the number density of T-cells with exhaustion value $s \in [0,1]$. We make the identifications:

$$ T(s,t) \sim n T_j(t) \qquad \text{ and } \qquad s \sim \frac{j}{n}, $$

where $s=0$ and $s=1$ correspond to fully-active and fully-exhausted T-cells, respectively. Defining $\epsilon = \frac{1}{n} \ll 1$, we recast the dimensionless discrete ODE model, defined by equations (15) and (16), in terms of the continuous variable *s*:

25 $$\begin{aligned} \frac{\partial T}{\partial t}(s,t) = \frac{1}{\epsilon } ( \kappa _0 + \kappa _1 N(t) ) \Bigl [ (1 - s + \epsilon )T(s - \epsilon , t) - (1 - s) T(s,t) \Bigr ] - \tilde{\gamma } T(s,t), \nonumber \\ \end{aligned}$$

for $\epsilon \le s \le 1$. We perform a Taylor series expansion of $T(s - \epsilon , t)$ in powers of the small parameter $\epsilon$. At leading order, we obtain a first-order hyperbolic partial differential equation for *T*(*s*, *t*):

26 $$\begin{aligned} \frac{\partial T}{\partial t} + \frac{\partial }{\partial s} \Bigl [ v(s,t) T \Bigr ] = - \tilde{\gamma } T, \end{aligned}$$

where the exhaustion velocity, *v*(*s*, *t*), is defined as follows:

27 $$\begin{aligned} v(s,t) = ( \kappa _0 + \kappa _1 N(t) )(1 - s) + \mathcal {O}(\epsilon ). \end{aligned}$$

We will refer to equations (26)–(27) as the (first-order) **PDE model**. In equations (26) and (27), we view *N*(*t*), the number of tumour cells, as a prescribed function of time *t* (in practice, *N*(*t*) is determined by first solving the reduced ODE model for *N*, $\Theta$ and $\mu$ defined by equations (11)–(13)).

We close equation (26) by prescribing initial and boundary conditions analogous to those used to solve the discrete ODE model. Specifically, we suppose that the T-cell population is initially at a steady state consistent with the absence of tumour cells:

28 $$\begin{aligned} T(s,0) = \frac{1}{\kappa _0} (1 - s)^{ - \left( 1 - \frac{\tilde{\gamma }}{\kappa _0} \right) }. \end{aligned}$$

We derive an appropriate boundary condition by making the following identification between *T*(0, *t*) and $T_0(t)$ in the continuum approximation of the discrete ODE model:

$$\begin{aligned} T(0,t) \sim \frac{1}{\epsilon }T_0(t). \end{aligned}$$

Then, equation (15) supplies:

$$\begin{aligned} \frac{\partial T}{\partial t}(0,t) = \frac{1}{\epsilon } \Biggl \{ 1 + \frac{ \tilde{\sigma } N(t)}{1 + \left( \frac{N(t)}{\tilde{N}} \right) ^2} - ( \kappa _0 + \kappa _1 N(t) ) T(0,t) \Biggr \} - \tilde{\gamma } T(0,t). \nonumber \end{aligned}$$

Since we seek bounded solutions in the limit as $\epsilon \rightarrow 0$, we prescribe:

29 $$\begin{aligned} T(0,t) = \frac{1}{\kappa _0 + \kappa _1 N(t)} \left( 1 + \frac{ \tilde{\sigma } N(t)}{1 + \left( \frac{N(t)}{\tilde{N}} \right) ^2} \right) . \end{aligned}$$

### Analytical Solutions

We use the method of characteristics to construct analytical solutions to the PDE model, defined by equations (26)–(29). In order to do this, it is convenient first to rewrite equation (26) in the following form:

30 $$\begin{aligned} \frac{\partial T}{\partial t} + (\kappa _0 + \kappa _1 N(t) )(1-s) \frac{\partial T}{\partial s} = ( \kappa _0 + \kappa _1 N(t) - \tilde{\gamma }) T. \end{aligned}$$

If we parameterise the characteristic curves by $\tau$, then the characteristic equations are given by:

31 $$\begin{aligned} \frac{dt}{d\tau } = 1, \qquad \frac{ds}{d\tau } = (\kappa _0 + \kappa _1 N(t))(1-s), \qquad \frac{dT}{d\tau } = (\kappa _0 + \kappa _1 N(t) - \tilde{\gamma } )T. \end{aligned}$$

**Fig. 10:**
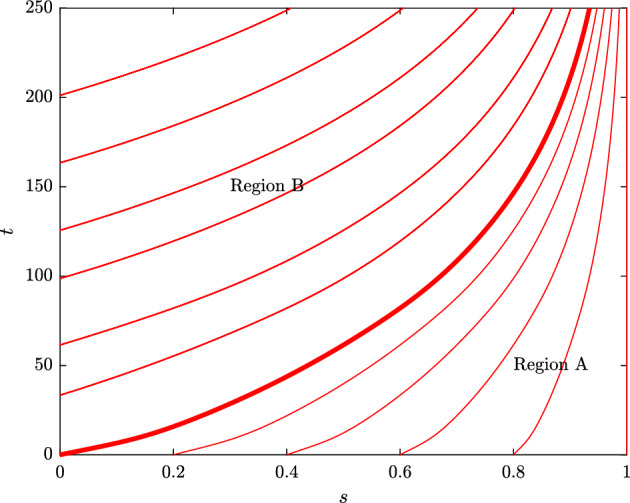
Characteristic projections. We plot the characteristic projections given by (31) in the (*s*, *t*) plane,. A discontinuity at (0, 0) propagates along the characteristic $s = S(t) = 1 - \text {exp}(- \int ^t_0 ( \kappa _0 + \kappa _1 N(u) ) \,du)$, dividing the plane into two regions, which we label A and B. Parameters are fixed at the default values listed in Table 2 (Color figure online)

We solve these characteristics in two distinct regions of the (*s*, *t*) plane, labelled A and B in Fig. 10; these regions are separated by the characteristic projection passing through $(s,t) = (0,0)$, which is given by, $s = S(t) :=1 - \text {exp}\Big (- \int ^t_0 \left( \kappa _0 + \kappa _1 N(u)\right) \,du \Big )$. In region A, the characteristic projections emanate from the initial conditions (28), and so we follow the trajectories of T-cells initially resident in the TME at $t =0$. In region B, the characteristic projections emanate from the boundary conditions (29), and so we follow the trajectories of T-cells recruited at $t > 0$. We note that as $t \rightarrow \infty$, *S*(*t*) tends to 1, and so at long times the T-cell exhaustion distribution is determined by the solution in region B, independent of the initial conditions.

We first solve in region A, where $S(t) < s \le 1$. For a characteristic projection passing through $(s,t) = (s_0, 0)$, we parameterise the initial conditions by:

$$\begin{aligned} t = 0, \quad s = s_0, \quad T = \frac{1}{\kappa _0} (1 - s_0)^{ - \left( 1 - \frac{\tilde{\gamma }}{\kappa _0} \right) }, \qquad \text {when } \tau = 0. \end{aligned}$$

Integrating characteristic equations (31) subject to these initial conditions, we obtain the parametric solution:

32 $$\begin{aligned} t(s_0, \tau )&= \tau , \end{aligned}$$

33 $$\begin{aligned} s(s_0, \tau )&= 1 - (1 - s_0) \text { exp} \left( - \int ^{\tau }_{0} \left( \kappa _0 + \kappa _1 N(u) \right) \,du \right) , \end{aligned}$$

34 $$\begin{aligned} T(s_0, \tau )&= \frac{1}{\kappa _0} (1 - s_0)^{ - \left( 1 - \frac{\tilde{\gamma }}{\kappa _0} \right) } \text {exp} \left( \int ^{\tau }_0 \left( \kappa _0 + \kappa _1 N(u) -\tilde{\gamma } \right) \,du \right) \end{aligned}$$

Eliminating variables $\tau$ and $s_0$, we obtain the explicit solution for *T*(*s*, *t*):

35 $$\begin{aligned} T(s,t) = \frac{1}{\kappa _0} (1 - s)^{ - \left( 1 - \frac{\tilde{\gamma }}{\kappa _0} \right) } \text {exp} \left( \int ^t_0 \frac{\tilde{\gamma } \kappa _1}{\kappa _0} N(u) \,du \right) , \end{aligned}$$

noting that this solution is valid where $S(t) = 1 - \text {exp}\Big (- \int ^t_0 \left( \kappa _0 + \kappa _1 N(u) \right) \,du \Big ) < s \le 1$.

We again use the method of characteristics to solve for *T*(*s*, *t*) in region B, where $0 \le s < S(t)$. We obtain a parametric solution for *T*(*s*, *t*) of the form:

36 $$\begin{aligned} T(s,t) = \frac{e^{- \tilde{\gamma } \big ( t - t_0 \big )}}{\kappa _0 + \kappa _1 N(t_0)} \left( 1 + \frac{ \tilde{\sigma } N(t_0)}{1 + \left( \frac{N(t_0)}{\tilde{N}} \right) ^2} \right) \frac{1}{(1-s)} , \end{aligned}$$

where $0< s < 1 - \text {exp}(- \int ^t_0 \left( \kappa _0 + \kappa _1 N(u) \right) \,du) = S(t)$, and $t_0 = t_0(s,t)$ is defined implicitly as follows:

37 $$\begin{aligned} s = 1 - \text {exp} \left( - \int ^{t}_{t_0(s,t)} \big ( \kappa _0 + \kappa _1 N(u) \big ) \,du \right) . \end{aligned}$$

The function $t_0(s,t)$ corresponds to the time at which T-cells, with exhaustion level *s* at time *t*, first infiltrated into the TME.

Equations (35) and (36)–(37) define the parametric solution to the PDE model, (26)–(29), and describe how the exhaustion distribution of a T-cell population changes over time. The characteristic $s = S(t)$, partitioning regions A and B, defines a wavefront of T-cells that propagates from left to right through exhaustion levels, as active T-cells are recruited to the TME and become exhausted due to the tumour. We note from equation (35) that in front of the wavefront ($S(t) < s \le 1$), the exhaustion profile is determined solely by the initial conditions, and the tumour growth dynamics determine whether the T-cells in this region grow exponentially or decay to zero. Taking the limit as $t \rightarrow \infty$ in equation (35), we find that the distribution in front of the wavefront decays to zero if $N^* < \frac{\tilde{\gamma } - \kappa _0}{\kappa _1}$ and becomes unbounded if $N^* > \frac{\tilde{\gamma } - \kappa _0}{\kappa _1}$. These results are consistent with steady state analysis of the discrete ODE model, in Sect. 3.2. Behind the wavefront ($0 \le s(t) < S(t)$), the exhaustion distribution is determined by the rate at which active T-cells are recruited to the TME, in response to the tumour. From equation (36), we note that there is a finite time delay, given by $t - t_0(s,t)$, due to the time it takes active T-cells recruited at time $t_0$ to attain an exhaustion level *s*. As the size of the tumour population, *N*(*t*), decreases, the time it takes for active T-cells to exhaust increases (i.e., $t - t_0(s,t)$ increases) and this causes the T-cell distribution to become skewed towards active T-cells.

When $\kappa _1 > 0$, it is not possible to construct an explicit expression for *T*(*s*, *t*) in region B. If we further make the assumption that the exhaustion rate is approximately constant, and set $\kappa _1 = 0$, then we obtain a fully explicit solution of the form:

38 $$\begin{aligned} T(s,t) = {\left\{ \begin{array}{ll} \frac{1}{\kappa _0} \left( 1 + \frac{ \tilde{\sigma } N\left( t + \frac{1}{\kappa _0} \text {ln}(1-s) \right) }{1 + \left( \frac{N\left( t + \frac{1}{\kappa _0} \text {ln}(1-s) \right) }{\tilde{N}} \right) ^2} \right) (1 - s)^{- \left( 1 - \frac{\tilde{\gamma }}{\kappa _0} \right) }, & \text {for } 0< s< 1 - e^{-\kappa _0 t}, \\ \frac{1}{\kappa _0} (1 - s)^{ - \left( 1 - \frac{\tilde{\gamma }}{\kappa _0} \right) }, & \text {for } 1 - e^{- \kappa _0 t}< s < 1. \end{array}\right. } \end{aligned}$$

### PDE Model Dynamics

In this section, we present analytical solutions to the first-order PDE model (26)–(29), given parametrically by equations (35)–(37), and compare them with solutions to the discrete ODE model (15)–(16). We first plot these results at a single time point ($t = 25$ days) for increasing values of *n* to demonstrate how the agreement between the PDE model and the discrete ODE model improves as $\epsilon = \frac{1}{n} \rightarrow 0$. We then present solutions to the PDE model at multiple time points ($t = 10, 25, 50, 100$ days) for different parameter values to illustrate the T-cell exhaustion distribution associated with the different qualitative behaviours that the model exhibits, consistent with the 3 E’s of immunoediting (Dunn et al. 2004).

**Fig. 11:**
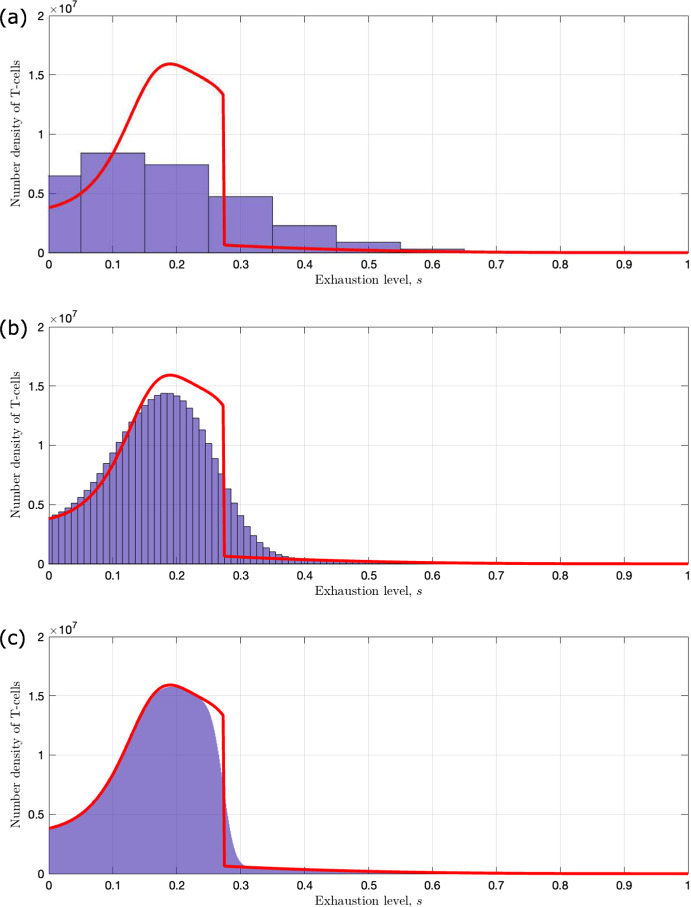
Analytical solutions of the first-order PDE model (26)–(29) show how its agreement with the discrete ODE model improves as $\epsilon = \frac{1}{n} \rightarrow 0$. We present analytical solutions to the first-order advection equation (26), given parametrically by equations (35)–(37), and compare them with numerical solutions to the discrete ODE model (15)–(16). We denote solutions to the PDE model in red and present solutions to the discrete model as blue histograms, for increasing values of *n*. We focus on a single time point ($t = 25$ days) for **a** $n = 10$, **b** $n = 100$, **c** $n = 1000$, to show how the accuracy of the Taylor series approximation improves as $\epsilon = \frac{1}{n} \rightarrow 0$. The tumour dynamics, *N*(*t*), used to solve the PDE model for *T*(*s*, *t*) are computed by solving the reduced ODE model (11)–(13), and are presented in Fig. 2b. The remaining parameters are fixed at the default values listed in Table 2, where $\tilde{\lambda } = 0.0442$ and $\kappa _1 = 1.39$ (Color figure online)

In Fig. 11, we show how the agreement between the PDE and discrete ODE models improves as we increase *n*, the number of discrete subgroups of exhaustion. Figure 11 shows that the PDE model provides a good approximation to the discrete ODE model, and captures the qualitative behaviour of a propagating wavefront of T-cells, that are driven through exhaustion levels as the tumour evolves. As *n* increases (and $\epsilon \rightarrow 0$), the accuracy of the Taylor series approximation increases. We note also that as *n* increases, the wavefront in the discrete ODE model solutions becomes steeper, with a highly concentrated wave peak. For smaller values of *n*, as in Fig. 11a, there is a greater discrepancy between the PDE model and discrete ODE model at the wavefront. Inclusion of $\mathcal {O}(\epsilon )$ terms reduces these differences (see Appendix B for more details).

**Fig. 12:**
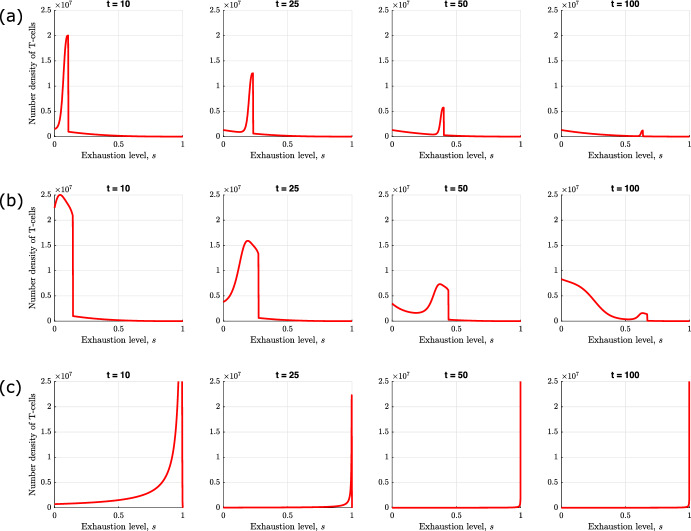
Analytical solutions of the first-order PDE model, as parameters $\tilde{\lambda }$ and $\kappa _1$ vary, showing the T-cell exhaustion dynamics for each of the different qualitative long-term behaviours of the model. We present analytical solutions to the first-order advection equation (26), given parametrically by equations (35)–(37), to show how the T-cell exhaustion profile evolves over time in different scenarios. We plot solutions to the PDE model in red, at times $t = 10, 25, 50, 100$ days. We present solutions for three sets of parameter values, for the rate of T-cell induced tumour kill, $\tilde{\lambda }$, and the exhaustion rate per tumour cell, $\kappa _1$: **a** when $\tilde{\lambda } = 0.29$ and $\kappa _1 = 1.39$ we observe tumour elimination; **b** when $\tilde{\lambda } = 0.0442$ and $\kappa _1 = 1.39$ we observe tumour equilibrium; **c** when $\tilde{\lambda } = 0.0442$ and $\kappa _1 = 80.0$ we observe tumour escape. The corresponding tumour dynamics, *N*(*t*), used to construct each of the PDE model solutions for *T*(*s*, *t*) are computed by solving the reduced ODE model (11)–(13), and are presented in Fig. 2. Except for $\tilde{\lambda }$ and $\kappa _1$, all parameters are fixed at the default values listed in Table 2 (Color figure online)

In Fig. 12, we present the temporal dynamics of the T-cell exhaustion distribution as $\tilde{\lambda }$ and $\kappa _1$ vary. In doing so, we demonstrate the different qualitative long-term behaviours that the model exhibits (see Fig. 2), consistent with the 3 E’s of immunoediting—tumour elimination, tumour equilibrium, and tumour escape—and how the T-cell exhaustion dynamics differ in each case. We recall that $\tilde{\lambda }$ corresponds to the rate of T-cell induced tumour kill and $\kappa _1$ corresponds to the rate of T-cell exhaustion per tumour cell. Initially, in all cases, a wave of T-cells propagates through exhaustion levels due to an initial influx of fully active T-cells, and the initial cytotoxic response to the tumour begins. In Fig. 12a, the cytotoxic efficacy of the T-cells is sufficient to readily eliminate the tumour. We observe a small, but sharp, wave peak that travels slowly, since the number of tumour cells is small and, therefore, the influx of T-cells recruited to the TME is small and they do not experience high levels of exhaustion. In Fig. 12b, the rate at which T-cells kills tumour cells is reduced (from $\tilde{\lambda } = 0.29$ to $\tilde{\lambda } = 0.0442$), and the T-cells cannot eliminate the tumour but they are able to manage the tumour burden until both populations reach an equilibrium. During these interactions, the T-cells must maintain a large active population to prevent the tumour from growing out of control. As such, we observe a larger, more diffuse wave of T-cells. In Fig. 12c, the rate of T-cell exhaustion per tumour cell is increased (from $\kappa _1 = 1.39$ to $\kappa _1 = 80.0$) and the wave of T-cell exhaustion propagates much more rapidly. The T-cells quickly accumulate at the exhausted phenotype and their reduced cytotoxic activity allows the tumour to escape the immune response. In Fig. 12c, the PDE model solution becomes unbounded as it approaches the boundary $s=1$. This is due to a singularity in the PDE model at $s=1$ where the Taylor-series approximation ceases to be valid and higher order terms become dominant.

## Discussion

In this paper, we have derived and analysed a mathematical model that focuses on how T-cell exhaustion impacts tumour growth and tumour-immune interactions in the TME. We have modelled a population of cytotoxic T-cells, structured by exhaustion level, and their co-evolution with a population of tumour cells. The T-cells become increasingly exhausted over time due to interactions with the tumour cells, which progressively decrease the rate of T-cell induced tumour kill. The model is well-mixed and governed by a system of time-dependent ODEs. We have shown that this model reduces to a closed system of ODEs describing the evolution of the total number of tumour cells, the total number of T-cells, and their mean exhaustion level. This reduced ODE model provides an alternative (coarse-grained) macroscale view of T-cell exhaustion dynamics, derived from a more fundamental description of the biology than existing models.

After deriving the model, we presented numerical simulations which show how, as key parameters characterising T-cell activity vary, the model transitions between different qualitative behaviours consistent with the 3 E’s of immunoediting—tumour elimination, tumour equilibrium, and tumour escape (Dunn et al. 2004). The solutions provide insight into how the distribution of T-cells across exhaustion states evolves during a tumour’s evolution and suggest that different T-cell distributions are associated with different tumour outcomes. We derived steady state solutions and characterised their linear stability to further understand the long-term behaviour of the model. We then presented bifurcation analysis which partitioned parameter space into distinct regions associated with each of these different qualitative long-term behaviours. We further located regions of parameter space where the model exhibits bistability, and a small range of parameter values for which the model exhibits tristability. Additionally, we incorporated treatment into the model to assess its impact on tumour-immune dynamics. Finally, we derived an equivalent PDE model of T-cell exhaustion dynamics, which is more analytically tractable than the discrete ODE model. The discrete ODE model, the reduced ODE model, and the PDE model provide different perspectives of T-cell exhaustion, at different levels of granularity, and, as such, provide complementary insights into T-cell exhaustion dynamics. Comparison of these models shows how parameters characterising T-cell exhaustion relate from the microscale to the macroscale.

The numerical solutions of the ODE models provided insight into T-cell exhaustion dynamics and their influence in determining the qualitative long-term behaviour of the model, consistent with the 3 E’s of immunoediting (Dunn et al. 2004). For both tumour elimination and equilibrium, we observed the mean exhaustion level remain low, since the tumour burden remains small and so T-cells undergo minimal levels of exhaustion. However, for tumour equilibrium we saw a much larger population of T-cells initially and at steady state than for tumour elimination, since the immune response remains active in order to prevent the tumour from growing any larger. In the case of tumour escape, we observed a highly exhausted population of T-cells, which leads to a lack of cytotoxic activity, allowing the tumour to grow to its full carrying capacity. These predictions suggest that analysing the exhaustion distribution of T-cells from patient samples could be used to predict whether a tumour will escape immune control and to personal immunotherapy for a given patient (i.e., identify which type of immunotherapy would be most beneficial for a particular patient).

The bifurcation analysis revealed that by changing key parameters that characterise T-cell activity, $\tilde{\lambda }$ and $\kappa _1$, we can transition from a large tumour steady state (tumour escape) to either a much smaller steady state (tumour equilibrium) or eliminate the tumour altogether (tumour elimination). Increasing the value of $\tilde{\lambda }$ in the model corresponds to increasing the efficiency of T-cell induced tumour kill. This could be achieved in the model by treatment with immunotherapeutic drugs designed to stimulate cytotoxic activity (Berraondo et al. 2019) or CAR-T cell therapy to introduce immune cells that are more efficient at locating and killing tumour cells (Sterner and Sterner 2021). Decreasing the value of $\kappa _1$ in the model corresponds to decreasing the rate of T-cell exhaustion per tumour cell. This change could be induced by administering immune checkpoint inhibitors that block negative immunoregulatory pathways such as PD-1 and CTLA-4 (Tabana et al. 2021), which are characteristically upregulated in exhausted T-cells. Therefore, the model shows that such immunotherapeutic strategies, that cause the desired changes to these model parameters, represent promising strategies to reduce tumour burden.

We showed that the PDE model provides a good approximation to the discrete ODE model, and captures the qualitative behaviour of the system: a propagating wavefront of T-cells driven through exhaustion levels by the tumour. The PDE model revealed an analytical solution that develops qualitative insight into T-cell exhaustion dynamics and demonstrates the roles of key parameters in determining the size and efficacy of the immune response. In practice, exhaustion in T-cells is identified by measuring the co-expression of multiple inhibitory receptors, which are continuously varying quantities. Therefore, the PDE model also offers a more natural framework for validating the model against T-cell exhaustion data than the discrete ODE model. We note that the PDE model exhibits singular behaviour at high exhaustion levels (as $s \rightarrow 1$). This is due to the functional form used to model exhaustion, which was chosen for analytical tractability. We could alter the functional form of the exhaustion function, but the resulting model would not, in general, reduce to a closed system of ODEs and would require numerical methods.

Although many existing models of tumour-immune interactions reproduce the 3 E’s of immunoediting, our models differ by providing insight into how the distribution of T-cell exhaustion may vary between the different tumour outcomes. This level of detail is not included in existing ODE models of tumour-immune interactions (Kuznetsov et al. 1994; Robertson-Tessi et al. 2012; Pillis and Radunskaya 2003). The reduced ODE model resembles the predator–prey models of Kuznetsov et al. (1994) and others, but incorporates the mean exhaustion-level of T-cells as an additional dependent variable, highlighting the effect of exhaustion on tumour-immune interactions. The reduced ODE model is derived from a structured model of exhaustion and, as such, better reflects current understanding of the biology than existing models, which focus on a smaller number of exhaustion states (Kareva and Gevertz 2024). A structured mathematical model of exhaustion also provides a natural framework for model validation using single-cell sequencing data. Additionally, by fitting to data describing the exhaustion profile of patients’ T-cells, the models could be used to identify those patients who would benefit from specific types of immunotherapy.

There are many ways in which our model could be extended in future work. For example, in the present model, the exhaustion-level of a cytotoxic T-cell only affects the rate at which it kills tumour cells and the rate at which it exhausts. In practice, however, cytotoxic T-cell exhaustion also affects proliferation and production of inflammatory cytokines, such as IFN$\gamma$ and TNF (Chow et al. 2022). The impact of these effects on the tumour’s growth dynamics could be incorporated into the model by allowing the source of T-cells to depend upon exhaustion level, and introducing additional dependent variables to account for specific, T-cell derived cytokines which promote immune-cell activity and/or inhibit tumour growth. In the present model, we focus on the role of cytotoxic T-cells in the immune response to cancer whereas, in practice, multiple immune cell populations play a key role. An interesting model extension would be to include a more detailed description of the immune system; we could distinguish between different T-cell subpopulations (e.g., helper T-cells, regulatory T-cells) and include other immune cell types (e.g., macrophages, B-cells, dendritic cells) and investigate how changes in their interactions may enable a tumour to evade immune control. The present model neglects spatial effects, and so an interesting model extension would be to account for spatial heterogeneity. Such a model could be used to investigate whether highly-exhausted T-cell niches form in close proximity to the tumour and whether this hampers immune activity and/or infiltration to the TME. Experiments by Zinselmeyer et al. suggest reduced motility in T-cells expressing exhaustion marker PD-1 (Zinselmeyer et al. 2013) and, therefore, a spatially-resolved model could investigate the additional impact of altered motility on the rate of tumour killing by exhausted T-cells. Another interesting extension would be to introduce stochastic effects into our model. This could capture randomness associated with T-cell exhaustion, such as in fluctuations in exhaustion marker expression and stochastic transitions between exhaustions states. Stochastic effects also have the potential to destabilize dynamic behaviour and so their inclusion would provide insight into the robustness of the model’s predictions (Lu et al. 2014). In the PDE model, stochastic effects would also regularise the discontinuity at the travelling wavefront.

Another, important direction for future work involves fitting the model to experimental data and validating its predictions. These data could comprise single-cell sequencing data taken at different time points during a tumour’s time course. By quantifying dynamic changes in the expression of exhaustion markers (e.g., PD-1 and CTLA-4), we could estimate how the exhaustion distribution of the T-cells changes over time. These data, combined with T-cell and tumour cell counts at each time point, could be fitted to the discrete ODE model, the reduced ODE model, and the PDE model. By fitting the model to single-cell sequencing data, the model could be used to identify patients who are likely to benefit from specific types of immunotherapy.

In conclusion, in this paper we have presented a structured mathematical model that investigates how T-cell exhaustion impacts tumour growth. The model reproduces the 3 E’s of immunoediting—elimination, equilibrium, and escape–and suggests how the qualitative form of the T-cell exhaustion distribution changes with tumour outcome. Our analysis shows that the model exhibits multi-stability, and that by altering key parameters we can drive the tumour from escape to elimination. The model illustrates how immunotherapies such as immune-checkpoint blockade, that mitigate the effects of exhaustion, can be used as effective treatments to reduce tumour burden.

## Figures and Tables

**Fig. 13:**
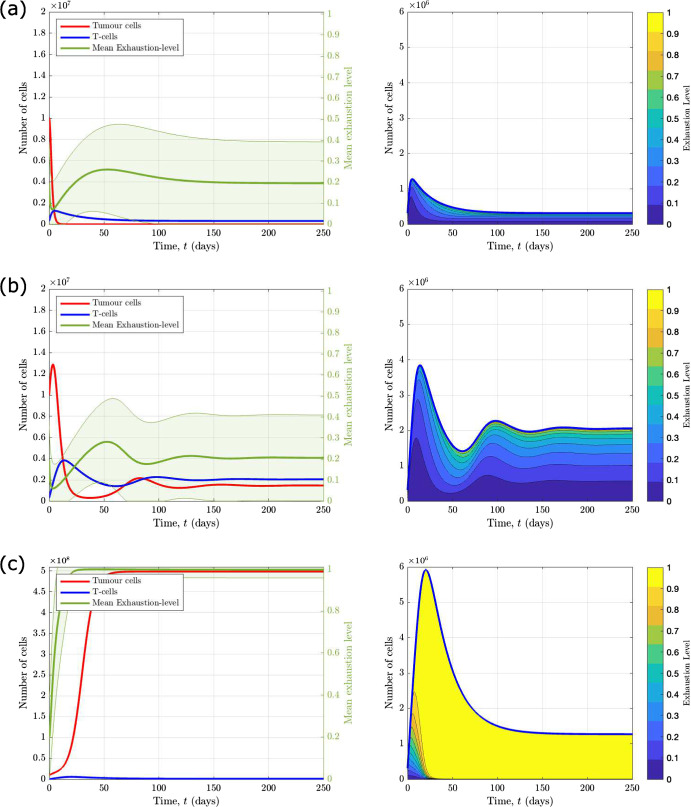
Numerical simulations illustrating the variance of the T-cell exhaustion distribution for different values of $\tilde{\lambda }$ and $\kappa _1$. On the left, we present solutions to equations (11)–(13) that show the evolution of the number of tumour cells (red), the total number of T-cells (blue), and their mean exhaustion level (green) over a period of 250 days. We compute solutions to equation (A1) for the variance, $\Sigma ^2$, and shade a green region one standard deviation from the mean exhaustion level, $[\mu - \Sigma , \mu + \Sigma ]$. On the right, we present solutions to equations (15)–(16) that show the evolution of the corresponding T-cell exhaustion distribution. We choose three sets of parameter values for the rate of T-cell induced tumour kill, $\tilde{\lambda }$, and the exhaustion rate per tumour cell, $\kappa _1$: **a** when $\tilde{\lambda } = 0.29$ and $\kappa _1 = 1.39$ we observe tumour elimination; **b** when $\tilde{\lambda } = 0.0442$ and $\kappa _1 = 1.39$ we observe tumour equilibrium; **c** when $\tilde{\lambda } = 0.0442$ and $\kappa _1 = 80.0$ we observe tumour escape. The remaining parameters are fixed at the default values stated in Table 2, with $n=10$ (Color figure online)

**Fig. 14:**
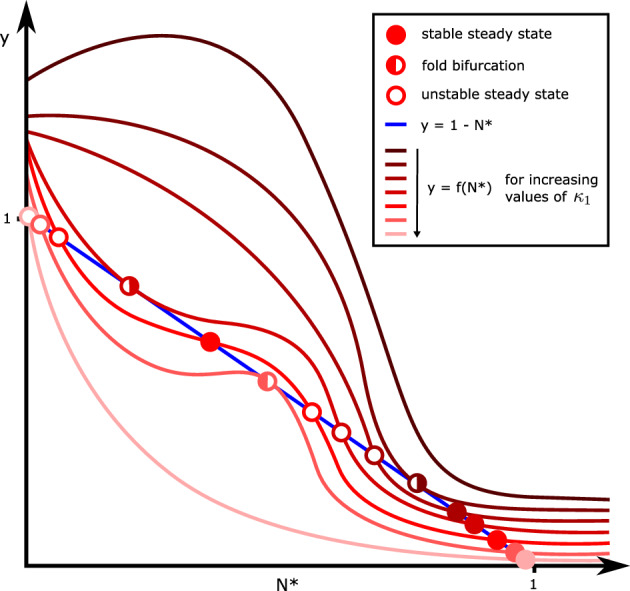
Schematic showing how intersections between the curves $y = f(N^*)$ and $y = 1 - N^*$ and, hence, the number of non-trivial solutions to equation (19) change as $\kappa _1$ varies, for the case $f(0) = \frac{\tilde{\lambda }}{\tilde{\gamma } + \kappa _0} > 1$. We sketch the curve $y = f(N^*)$ (red) for 7 different values of $\kappa _1$ (the rate of T-cell exhaustion per tumour cell), to illustrate the different qualitative ways in which it intersects with the line $y = 1 - N^*$ (blue). The points of intersection are indicated by circles: filled circles represent stable steady state solutions; unfilled circles represent unstable steady state solutions; half-filled circles represent fold bifurcations, where a stable and unstable steady state coalesce/emerge. We assume $f(0) = \frac{\tilde{\lambda }}{\tilde{\gamma } + \kappa _0} > 1$, in which case equation (19) has either zero, two, or four non-trivial solutions (Color figure online)

**Fig. 15:**
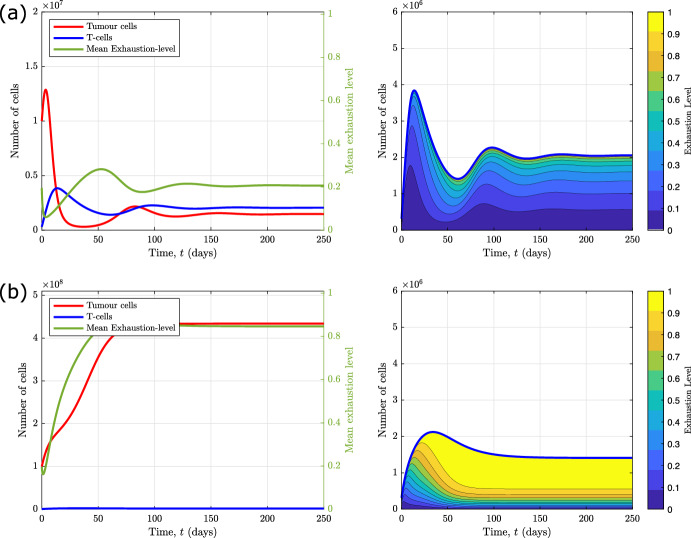
Demonstration of the coexistence of stable steady states for $\tilde{\lambda } = 0.0442$ and $\kappa _1 = 1.39$. As in Fig. 2, on the left we present solutions to equations (11)–(13) that show the evolution of the number of tumour cells (red), the total number of T-cells (blue), and their mean exhaustion level (green) over a period of 250 days. On the right, we present solutions to equations (15)–(16) that show the evolution of the corresponding T-cell exhaustion distribution. Parameters are fixed at the default values stated in Table 2, with $n=10$, but we vary the initial conditions of the tumour, $\hat{N}(0) = N_0$. For these parameter values, the system is bistable and evolves to different steady states depending on the initial conditions: **a** for $N_0 = 10^7$ cells, we observe tumour equilibrium; **b** for $N_0 = 10^8$ cells, we observe tumour escape (Color figure online)

**Fig. 16:**
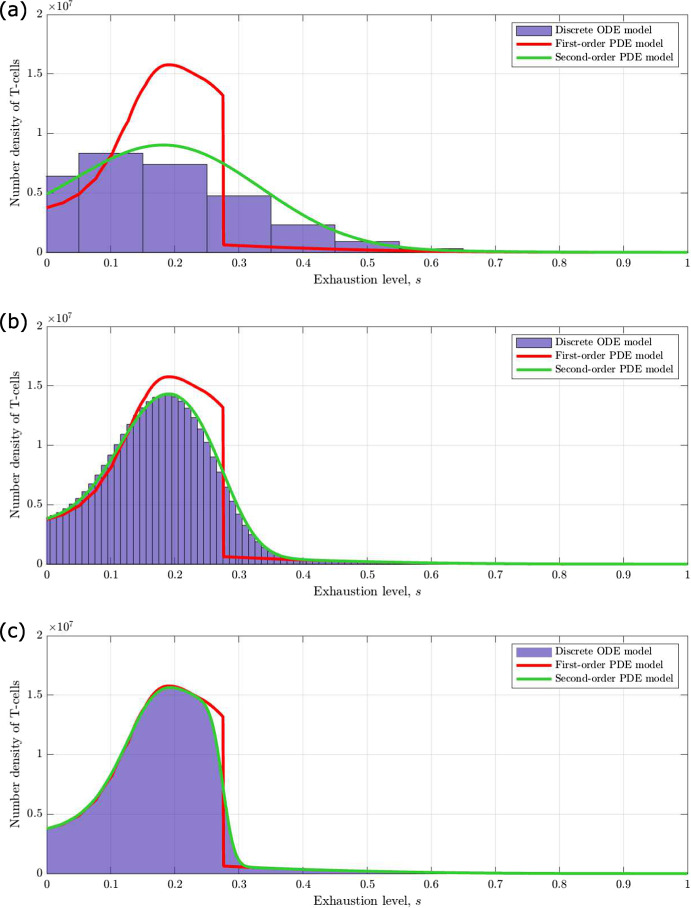
Comparison of solutions to the second-order PDE model with solutions to the discrete ODE model and the first-order PDE model, as $\epsilon = \frac{1}{n} \rightarrow 0$. We present numerical solutions to the advection–diffusion equation (B8), describing the evolution and exhaustion of the T-cell population, solved using the method of lines. We plot the solutions of the second-order PDE model (B8)–(B12) in green and compare with solutions of the first-order PDE model (26)–(29) in red and solutions of the discrete ODE model (15)–(16) in blue. We present these figures at a single time point ($t = 25$ days) for **a** $n = 10$, **b** $n = 100$, **c** $n = 1000$, to show how the accuracy of the first- and second-order Taylor series approximation compare as $\epsilon = \frac{1}{n} \rightarrow 0$. The corresponding tumour dynamics, *N*(*t*), used to construct the PDE model solutions for *T*(*s*, *t*) are computed by solving the reduced ODE model (11)–(13), and are presented in Fig. 2b. The remaining parameters are fixed at the default values listed in Table 2, where $\tilde{\lambda } = 0.0442$ and $\kappa _1 = 1.39$ (Color figure online)

## Data Availability

The code used in the current study is available on request from the corresponding author.

## Further Analysis of the ODE Model

### Variance of the T-cell Exhaustion Distribution

We can use equations (1)–(2) and (7)–(8) to derive an expression for the variance of the T-cell exhaustion distribution, $\Sigma ^2$. Explicitly, we define $\Sigma ^2$, in terms of non-dimensional variables, as:

$$\begin{aligned} \Sigma ^2 = \frac{1}{\Theta } \sum _{j=0}^n \left( \frac{j}{n} \right) ^2 {T}_j \, - \mu ^2. \end{aligned}$$

Differentiating this expression with respect to time *t*, and substituting from equations (15)–(16) and (11)–(12), we obtain the following non-dimensional ODE for $\Sigma ^2$:

A1 $$\begin{aligned} \frac{d\Sigma ^2}{dt}=(\kappa _0 + \kappa _1 N) \frac{1}{n} (1 - \mu ) - \frac{\Sigma ^2 -\mu ^2}{\Theta } \left( 1 + \frac{\tilde{\sigma } N}{1 + \left( \frac{N}{\tilde{N}} \right) ^2}\right) - 2 (\kappa _0 + \kappa _1 N) \Sigma ^2. \nonumber \\ \end{aligned}$$

Let ${\Sigma ^2}^*$ be the steady state solution of the variance of the T-cell distribution. Setting $\frac{d}{dt} = 0$ in equation (A1) and substituting in expressions for $\Theta ^*$ and $\mu ^*$ (18), we obtain the following expression for ${\Sigma ^2}^*$:

A2 $$\begin{aligned} {\Sigma ^2}^* = \frac{\mu ^* (1 - \mu ^*)}{1 + \mu ^*} \left( \frac{1}{n} + \mu ^* \right) \end{aligned}$$

where we write $\Sigma ^2$ in terms of the mean exhaustion-level steady state, $\mu ^*$, and the number of the discrete exhaustion levels, *n*. In Fig. 13, we plot the numerical solutions of equations (11)–(13) from Sect. 3.1, and include the corresponding variance of the exhaustion distribution by solving equation (A1). We shade a region in green that is one standard deviation from the mean exhaustion level (i.e., $[\mu - \Sigma , \mu + \Sigma ]$).

### Steady State Analysis

As shown in Sect. 3.3, we deduce that the reduced ODE model (11)–(13) possesses at most 5 steady state solutions by considering intersections of the curve $y = f(N^*)$ with the line $y = 1 - N^*$ as solutions to equation (19). In Fig. 14, we show a graphical representation of equation (19) for $\frac{\tilde{\lambda }}{\tilde{\gamma } + \kappa _0} > 1$. In Fig. 15, we present two numerical solutions which, for the same parameter values but different initial conditions, evolve to different steady state, demonstrating the coexistence of stable steady state solutions.

### Linear Stability Analysis

We seek solutions to the governing equations (11)–(13) of the form:

A3 $$\begin{aligned} \Theta (t) \approx \Theta ^* + \delta \Theta _p(t), \quad \mu (t) \approx \mu ^* + \delta \mu _p(t), \quad N(t) \approx N^* + \delta N_p(t), \end{aligned}$$

where $0 < \delta \ll 1$ is a small parameter. By substituting from (A3) into equations (11)–(13), and equating terms of $\mathcal {O}(\delta )$, we obtain the linearised system $\frac{d {\textbf {W}}}{dt} = {\textbf {J}} {\textbf {W}}$, where ${\textbf {W}} = (\Theta _p, \mu _p, N_p)^T$ and ${\textbf {J}}$ denotes the following Jacobian matrix:

A4 $$\begin{aligned} {\textbf {J}} = \begin{bmatrix} -\tilde{\gamma } & 0 & \sigma '(N^*) \\ \frac{\tilde{\gamma }^2 k(N^*)}{\sigma (N^*)(k(N^*) + \tilde{\gamma })} & -(k(N^*) + \tilde{\gamma }) & \frac{k(N^*) \tilde{\gamma }}{k(N^*) + \tilde{\gamma }} \left( \frac{k'(N^*)}{k(N^*)} - \frac{\sigma '(N^*)}{\sigma (N^*)} \right) \\ -\frac{\tilde{\lambda }\tilde{\gamma }N^*}{k(N^*) + \tilde{\gamma }} & \frac{\tilde{\lambda }}{\tilde{\gamma }} N^* \sigma (N^*) & (1-2N^*) - \frac{\tilde{\lambda }\sigma (N^*)}{k(N^*) + \tilde{\gamma }} \end{bmatrix}, \end{aligned}$$

where $'$ denotes differentiation with respect to *N*. We write $k(N) = \kappa _0 + \kappa _1 N$ and $\sigma (N) = 1 + \frac{ \tilde{\sigma } N}{1 + \left( \frac{N}{\tilde{N}} \right) ^2}$, and *f*(*N*) as defined in equation (19).

We consider solutions of the form ${\textbf {W}} = \hat{{\textbf {W}}}e^{\alpha t}$, where the eigenvalue $\alpha$ satisfies:

A5 $$\begin{aligned} \text {det}({\textbf {J}} - \alpha {\textbf {I}}) = - (\alpha + \tilde{\gamma }) \Bigl [ (\alpha + \tilde{\gamma } + k(&N^*) ) \left( \alpha + f(N^*) - (1-2N^*) \right) \nonumber \\&- N^* \Big ( f(N^*) k'(N^*) - \tilde{\lambda }\sigma '(N^*) \Big ) \Bigr ] = 0. \end{aligned}$$

A steady state is stable if Re($\alpha ) < 0$ for all eigenvalues $\alpha$. For the tumour-free steady state, $N^* = 0$, we have:

$$ \alpha _1 = - \tilde{\gamma }, \quad \alpha _2 = - (\tilde{\gamma } + \kappa _0), \quad \alpha _3 = 1 - f(0). $$

Since $\tilde{\gamma }, \kappa _0 > 0$, we deduce that $N^* = 0$ is unstable if $f(0) = \frac{\tilde{\lambda }}{\tilde{\gamma } + \kappa _0} < 1$, and linearly stable otherwise.

For physically realistic, non-trivial steady state solutions, we have $N^* > 0$ and $f(N^*) = 1 - N^*$ (19). Therefore, substituting (19) into (A5), we have that:

$$ - (\alpha + \tilde{\gamma }) \Bigl [ \alpha ^2 + (\tilde{\gamma } + k(N^*) + N^*) \alpha - N^* g(N^*) \Bigr ] = 0, $$

where the function $g(N^*)$ is defined as follows:

$$ g(N^*) :=( 1 - N^*) k'(N^*) - \tilde{\lambda }\sigma '(N^*) - \tilde{\gamma } - k(N^*). $$

Since $\tilde{\gamma } + k(N^*) +N^* > 0$, the non-trivial steady state is unstable if $g(N^*) > 0$, and stable if $g(N^*) < 0$. Expanding the exhaustion and influx functions, the steady state is unstable if,

A6 $$\begin{aligned} g(N^*) = \kappa _1 (1 - N^*) - \tilde{\lambda } \tilde{\sigma } \frac{1 - \left( \frac{N^*}{\tilde{N}} \right) ^2}{\left( 1 + \left( \frac{N^*}{\tilde{N}} \right) ^2 \right) ^2 } - \tilde{\gamma } - \kappa _0 - \kappa _1 N^* > 0. \end{aligned}$$

We now show that the stability of the T-cell distribution steady state (20) depends upon the same condition by linearising the full model. Let ${\textbf {V}} = (T_{0_p}, \dots , T_{n_p}, \Theta _p, \mu _p, N_p )$ be perturbations about the steady state. Then $\frac{d {\textbf {V}}}{dt} = {\textbf {J}}_1 {\textbf {V}}$ where ${\textbf {J}}_1$ is the following Jacobian matrix:

$$ {\textbf {J}}_1 = \begin{bmatrix} {\textbf {A}} & {\textbf {B}} \\ {\textbf {0}} & {\textbf {J}} \end{bmatrix},$$

where **J** is as defined in equation (A4), and **A** and **B** are matrices to be determined.

We consider solutions of the form ${\textbf {V}} = \hat{{\textbf {V}}}e^{\alpha t}$, where $\alpha$ is such that det(${\textbf {J}}_1 - \alpha {\textbf {I}}$) = det$({\textbf {A}} - \alpha {\textbf {I}})$det$({\textbf {J}} - \alpha {\textbf {I}})$ = 0. It is trivial to show that ${\textbf {A}}$ is a lower triangular matrix and, therefore, its determinant is the product of its diagonal, given by:

$$ \text {det}({\textbf {A}} - \alpha {\textbf {I}}) = \prod _{i = 0}^n \Big [ -(n - i)(\kappa _0 + \kappa _1 N^*) - \tilde{\gamma } - \alpha \Big ] $$

.

${\textbf {A}}$ has eigenvalues $\alpha _i = -(n - i)(\kappa _0 + \kappa _1 N^*) - \tilde{\gamma } < 0$ for $i = 0, \dots , n,$ and so for Re$(\alpha ) > 0$ we require det$({\textbf {J}} - \alpha {\textbf {I}}) = 0$. Therefore, the discrete ODE model steady state has the same linear stability as the reduced ODE model steady state.

## Second-Order PDE Model

### Advection–Diffusion Equation

Let *T*(*s*, *t*) be the non-dimensional number density of T-cells with exhaustion value, $s \in [0,1]$. We make the identifications:

$$ T(s,t) \sim n T_j(t) \qquad \text{ and } \qquad s \sim \frac{j}{n}, $$

where $s=0$ and $s=1$ correspond to fully-active and fully-exhausted T-cells, respectively. Defining $\epsilon = \frac{1}{n} \ll 1$, we recast the discrete ODE model, defined by Equations (15), (16), in terms of continuous variables *s* and *t*:

$$ \frac{\partial T}{\partial t}(s,t) = \frac{1}{\epsilon } ( \kappa _0 + \kappa _1 N(t) ) \Bigl [ (1 - s + \epsilon )T(s - \epsilon , t) - (1 - s) T(s,t) \Bigr ] - \tilde{\gamma } T(s,t) $$

for $\epsilon \le s \le 1$. Taylor expanding $T(s - \epsilon , t)$ in terms of $\epsilon$, we get:

B7 $$\begin{aligned} \frac{\partial T}{\partial t}(s,t) = ( \kappa _0 + \kappa _1 N(t) ) \Bigl [ -\frac{\partial }{\partial s} + \frac{\epsilon }{2} \frac{\partial ^2}{\partial s^2} + \mathcal {O}(\epsilon ^2) \Bigr ] \bigr [ (1-s)T(s,t) \bigl ] - \tilde{\gamma } T(s,t). \nonumber \\ \end{aligned}$$

We identify this as an advection–diffusion equation. Retaining terms of $\mathcal {O}(\epsilon )$, this expression can be written in the following, equivalent, conservation form:

B8 $$\begin{aligned} \frac{\partial T}{\partial t} = \frac{\partial }{\partial s} \Bigl [ D(s,t) \frac{\partial T}{\partial s} - v(s,t) T \Bigr ] - \tilde{\gamma } T, \end{aligned}$$

where the exhaustion velocity, *v*, and diffusion coefficient, *D*, are defined as follows:

B9 $$\begin{aligned} D(s,t) = \frac{\epsilon }{2}( \kappa _0 + \kappa _1 N(t) )(1 - s) \quad \text{ and } \quad v(s,t) = ( \kappa _0 + \kappa _1 N(t) )(1 - s) + \frac{\epsilon }{2}( \kappa _0 + \kappa _1 N(t) ). \end{aligned}$$

In equations (B8) and (B9), we view *N*(*t*) as a prescribed function of time *t*, determined by solving equations (11)–(12).

We suppose that the T-cell population is initially at a tumour-free steady state, and so prescribe the following initial condition:

B10 $$\begin{aligned} T(s,0) = \frac{1}{\kappa _0} (1 - s)^{ - \left( 1 - \frac{\tilde{\gamma }}{\kappa _0} \right) }. \end{aligned}$$

We close the second-order PDE model by prescribing the flux of T-cells at both ends of the domain:

B11 $$\begin{aligned} \Bigl [ v(s,t) T - D(s,t) \frac{\partial T}{\partial s} \Bigr ]_{s=0}&= 1 + \frac{ \tilde{\sigma } N(t)}{1 + \left( \frac{N(t)}{\tilde{N}} \right) ^2}, \end{aligned}$$

B12 $$\begin{aligned} \Bigl [ v(s,t) T - D(s,t) \frac{\partial T}{\partial s} \Bigr ]_{s=1}&= 0. \end{aligned}$$

### Second-Order PDE Model Dynamics

In Fig. 16, we present numerical solutions to the second-order PDE model of T-cell exhaustion dynamics, given by equations (B8)–(B12), and compare with numerical solutions to the discrete ODE model (15)–(16) and analytical solutions to the first-order PDE model (26)–(29). We present these solutions for three different sets of parameter values for $\tilde{\lambda }$ and $\kappa _1$ to illustrate how the T-cell distribution evolves for each of the different qualitative behaviours that the model exhibits. Figure 16 shows that the second-order PDE model replicates the discrete ODE model better than the first-order PDE model, particularly for smaller values of *n*, but the solutions rely on numerical methods and so are less interpretable.
